# Genome-wide identification and characterization of the SBP-box gene family in *Petunia*

**DOI:** 10.1186/s12864-018-4537-9

**Published:** 2018-03-12

**Authors:** Qin Zhou, Sisi Zhang, Feng Chen, Baojun Liu, Lan Wu, Fei Li, Jiaqi Zhang, Manzhu Bao, Guofeng Liu

**Affiliations:** 1 Key Laboratory of Horticultural Plant Biology, Ministry of Education; Key Laboratory of Urban Agriculture in Central China, Ministry of Agriculture; College of Horticulture and Forestry Sciences, Huazhong Agricultural University, Shizishan Street No. 1, Wuhan, 430070 China; 2 Wuhan Institute of Landscape Architecture, Peace Avenue No. 1240, Wuhan, 430081 China

**Keywords:** *Petunia*, *SPL* genes, Transcription factor, Expression patterns, miR156/miR157, Flowering

## Abstract

**Background:**

SQUAMOSA PROMOTER BINDING PROTEIN (SBP)-box genes encode a family of plant-specific transcription factors (TFs) that play important roles in many growth and development processes including phase transition, leaf initiation, shoot and inflorescence branching, fruit development and ripening etc. The SBP-box gene family has been identified and characterized in many species, but has not been well studied in *Petunia*, an important ornamental genus.

**Results:**

We identified 21 putative *SPL* genes of *Petunia axillaris* and *P. inflata* from the reference genome of *P. axillaris* N and *P. inflata* S6, respectively, which were supported by the transcriptome data. For further confirmation, all the 21 genes were also cloned from *P. hybrida* line W115 (Mitchel diploid). Phylogenetic analysis based on the highly conserved SBP domains arranged *PhSPLs* in eight groups, analogous to those from *Arabidopsis* and tomato. Furthermore, the *Petunia SPL* genes had similar exon**-**intron structure and the deduced proteins contained very similar conserved motifs within the same subgroup. Out of 21 *PhSPL* genes, fourteen were predicted to be potential targets of PhmiR156/157, and the putative miR156/157 response elements (MREs) were located in the coding region of group IV, V, VII and VIII genes, but in the 3’-UTR regions of group VI genes. *SPL* genes were also identified from another two wild *Petunia* species, *P. integrifolia* and *P. exserta*, based on their transcriptome databases to investigate the origin of *PhSPLs*. Phylogenetic analysis and multiple alignments of the coding sequences of *PhSPLs* and their orthologs from wild species indicated that *PhSPLs* were originated mainly from *P. axillaris*. qRT-PCR analysis demonstrated differential spatiotemperal expression patterns of *PhSPL* genes in petunia and many were expressed predominantly in the axillary buds and/or inflorescences. In addition, overexpression of *PhSPL9a* and *PhSPL9b* in *Arabidopsis* suggested that these genes play a conserved role in promoting the vegetative-to-reproductive phase transition.

**Conclusion:**

Petunia genome contains at least 21 *SPL* genes, and most of the genes are expressed in different tissues. The *PhSPL* genes may play conserved and diverse roles in plant growth and development, including flowering regulation, leaf initiation, axillary bud and inflorescence development. This work provides a comprehensive understanding of the SBP-box gene family in *Petunia* and lays a significant foundation for future studies on the function and evolution of *SPL* genes in petunia.

**Electronic supplementary material:**

The online version of this article (10.1186/s12864-018-4537-9) contains supplementary material, which is available to authorized users.

## Background

Transcription factors (TFs) are essential for the regulation of precise and coordinate gene expression via activating and/or repressing transcription in response to various endogenous and environmental signals, which plays crucial roles in the regulation of gene networks for many important developmental processes and defense responses in plants. TFs are usually classified into different families based on the sequence of DNA-binding domains and other conserved motifs [1]. SQUAMOSA PROMOTER BINDING PROTEIN (SBP)-box genes encode a family of plant-specific TFs that contain a highly conserved DNA-binding domain of 76 amino acids called the SBP domain [2, 3], which can form two tandem zinc fingers (Cys-Cys-His-Cys and Cys-Cys-Cys-His), and possesses a nuclear localization signal (NLS) at the C-terminus partially overlapping with the second zinc finger [4, 5]. The SBP domain is involved in both nuclear import and binding to a consensus DNA sequence with a GTAC core motif and gene-specific flanking regions [6, 7].

AmSBP1 and AmSBP2 are the first SBP-domain proteins discovered in plants, which were isolated from *Antirrhinum majus* in an in vitro approach and named after their binding ability to the promoter of the floral meristem identity gene *SQUAMOSA* [2]. Since then, SBP-box genes have been found in diverse plant species, including single-celled green algae, mosses, gymnosperms, and angiosperms [8]. Furthermore, genome-wide identified and analysis of the whole gene family were reported recently in some species, such as *Arabidopsis* [9, 10], rice [3], tomato [11], grape [12], apple [13], castor bean [14], *Populus* [15], *Citrus* [16], *Gossypium* [17], pepper [18], tobacco [19], oilseed rape [20], soybean [21], and moso bamboo [22]. In *Arabidopsis*, there are 16 *SBP-LIKE* (*SPL*) genes, which can be categorized into eight clades based on the amino acid sequence of their conserved SBP domain: *AtSPL1/12/14/16*, *AtSPL2/10/11*, *AtSPL3/4/5*, *AtSPL6*, *AtSPL7, AtSPL8, AtSPL9/15*, and *AtSPL13* [3, 8]. Among them, ten genes from five clades (*AtSPL2/10/11*, *AtSPL3/4/5*, *AtSPL6*, *AtSPL9/15* and *AtSPL13*) are targeted by miR156 [3, 23], an evolutionarily highly conserved miRNA in plants that plays master function of regulating the transition from the juvenile to the adult phase of vegetative development (vegetative phase change) [24, 25].

*SPL* genes control many aspects of plant development and physiology, including the vegetative phase change, flowering time, leaf initiation, shoot and inflorescence branching, fruit development and ripening, floral organ development and fertility, pollen sac development, trichome development, root development, and stress responses [8, 9, 26–28]. Recent studies provide a detailed picture of the function of miR156-targeted *SPL* genes in *Arabidopsis*: *AtSPL2/9/10/11/13/15* contribute to both the juvenile-to-adult phase transition (vegetative phase change) and the vegetative-to-reproductive phase transition (reproductive phase change or flowering), with *AtSPL9*/*13/15* being more important than *SPL2/10/11* [9]; *AtSPL3/4/5* were previously reported to redundantly promote flowering through direct activation of *LEAFY* (*LFY*), *FRUITFUL* (*FUL*), and *APETALA1* (*AP1*) [29], and they may act synergistically with the *FLOWERING LOCUS T* (*FT*)-*FD* module to induce flowering under long-day (LD) condition [30], whereas a recent study suggested that *AtSPL3/4/5* don’t play a major role in vegetative phase change or flowering, but promote the floral meristem identity transition [9]; *AtSPL6* does not have a major function in vegetative morphogenesis [9], it can positively regulate a subset of defense genes, however, and plays a role in effector-triggered immunity [31]. Among the miR156 non-targeted *SPL* genes, *AtSPL7* is a central regulator of copper (Cu) homeostasis and play a major role in cadmium (Cd) response [32, 33]. *AtSPL8* was found to be a tissue-dependent regulator in GA signaling and regulate early anther development and gynoecium differential patterning in concert with multiple miR156 targeted *SPL* genes, as well as trichome formation on sepals, stamen filament elongation, and root growth [34–37]. *AtSPL14* gene may function to delay phase transition [38].

Studies in other species revealed that *SPL* genes underwent an extensive neofunctionalization during their evolution. For instance, the maize *TEOSINTE GLUME ARCHITECTURE1* (*tga1*) gene, an *AtSPL13* homolog, controls glume development [39]. The rice *TGA1* ortholog *GW8/OsSPL16* gene controls grain size, shape and quality [40, 41]. The maize *LIGULELESS1* (*LG1*) gene (closest to *AtSPL8*) and its ortholog in rice (*OsLG1*) were found to control the formation of the ligule and auricule [42, 43]. Recently, they were found to also control the branch angle of tassel and panicle, respectively [44–46]. The tomato *Colorless non-ripening* (*Cnr*) locus encode an *AtSPL3* ortholog and is essential for fruit ripening [47].

Petunia (*Petunia hybrida*) is a popular and important ornamental species belonging to the economically important family Solanaceae that has been used as a genetic model system for a long history [48]. Petunia is also a good model for comparative research of gene function become of its many advantages, including the evolutionary position of asterid, ease of cultivation and propagation, highly efficient genetics and transformation. However, our knowledge on the evolution and function of *SBP*-box gene family in petunia is very limited yet. Only one recent study functionally characterized two petunia *SPL* genes, *PhSBP1* and *PhSBP2*, which showed that they have overlapping but divergent functions in the reproductive transition and leaf initiation rate [49]. Recently, the whole-genome sequencing was achieved for inbred derivatives of two wild *Petunia* species, *P. axillaris* N and *P. inflata* S6 [50], which provides a good opportunity to study the important gene family of TFs on a genome-wide scale. *P. hybrida* was considered to be originated from the hybridization of two biological species, one with erect habit and white flowers (*P. axillaris*), and the other with decumbent stems and purple flowers (*P. integrifolia*). *P. inflata* was previously considered as a synonym of *P. integrifolia*, and subsequently as a subspecies under *P. integrifolia* (*P. integrifolia* Subs*p. inflata*), but a recent study accepted it as distinct from *P. integrifolia* with the unfolded and straight calyx lobes as useful diagnostic characters [51]. Here, we report a genome- and transcriptome-wide analysis of the SBP-box gene family in *Petunia*, including *P. axillaris*, *P. inflata*, *P. integrifolia*, *P. hybrida*, and *P. exserta*, a unique species in the genus, with red corolla and distinct exserted stamens and stigma. Based on genome and transcriptome scanning, 21 SBP-box genes were identified in *P. axillaris* and *P. inflata*, respectively, confirmed by cloning from *P. hybrida* line W115. The gene structure, phylogeny, motif composition, miRNA target site and expression pattern in various tissues were systematically analyzed. In addition, the function of two representative genes, *PhSPL9a* and *PhSPL9b*, was characterized in transgenic *Arabidopsis* plants. The data presented here provide good foundation for understanding the key roles of *SPL* genes in petunia development and other biological processes.

## Results

### Identification of *Petunia SPL* genes

The *SPLs* of Arabidopsis, tomato and tobacco were used to search the genome and transcriptome datasets of *P. axillaris* and *P. inflata* [50, 52], which identified 21 putative *SPL* genes in the two species, designated as *PaSPLs* and *PiSPLs* (Additional files 1 and 2), respectively. To validate the full-length open reading frame (ORF) sequences and predicted exon-intron structures of the *Petunia SPL* genes, the coding sequence of *PhSPL* genes (*PhSPLs*) were cloned by RT-PCR from *P. hybrida* line W115. As a result, all 21 *SPL* genes were isolated successfully from W115 and confirmed by sequencing. We named these genes according to the closest homologs of them in Arabidopsis (Table 1).

**Table 1:** Nomenclature of *SPL* genes in *Petunia hybrida* line W115 (Mitchel diploid)

Gene name	Accession number	Preston et al. [49]	CDS length (bp)
*PhSPL2*	MF580469		1404
*PhCNR*	MF580470	*PhCNR*	285
*PhSPL3*	MF580471	*PhSBP1*	423
*PhSPL4a*	MF580472		420
*PhSPL4b*	MF580473		636
*PhSPL4c*	MF580474	*PhSBP2*	537
*PhSPL6a*	MF580475		1575
*PhSPL6b*	MF580476		1491
*PhSPL6c*	MF580477		1563
*PhSPL6d*	MF580478		1539
*PhSPL6e*	MF580479		1527
*PhSPL7*	MF580480		2406
*PhSPL8*	MF580481		915
*PhSPL9a*	MF580482		1164
*PhSPL9b*	MF580483		1125
*PhSPL9c*	MF580484		1146
*PhSPL12a*	MF580485		3021
*PhSPL12b*	MF580486		2958
*PhSPL12c*	MF580487		2910
*PhSPL12d*	MF580488		3027
*PhSPL13*	MF580489		990

### Phylogenetic analysis of SPL proteins from petunia, tomato, and *Arabidopsis*

**Fig. 1:**
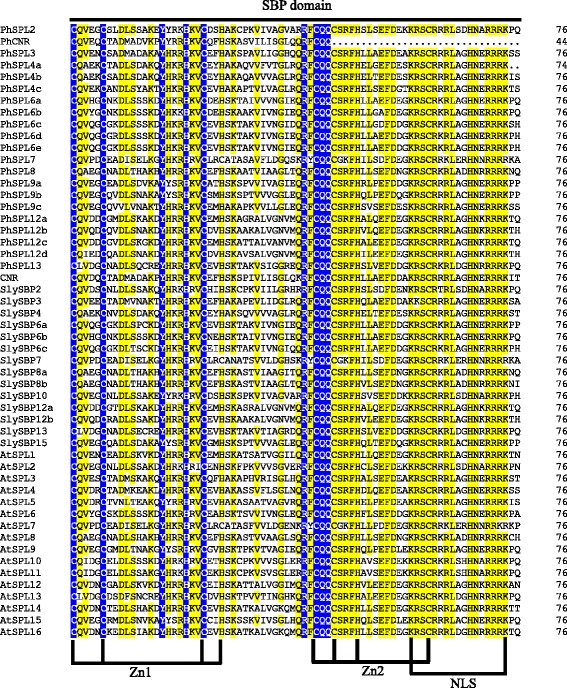
Multiple alignments of the highly conserved SBP-domains. Multiple alignments of the highly conserved SBP-domains (76 aa) of 21 PhSPL proteins, 15 SlySBP proteins, and 15 AtSPL proteins. The conserved SBP domain included 2 Zn-finger structures and NLS

**Fig. 2:**
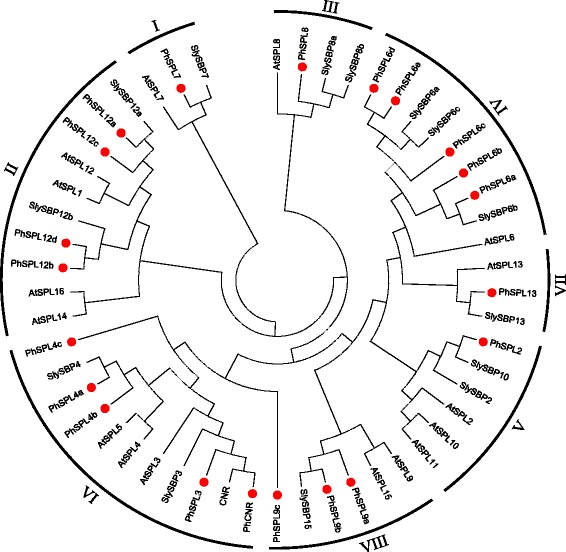
Phylogenetic tree based on the conserved SBP domains. Phylogenetic tree based on the conserved SBP domains (76aa) alignment of 21 PhSPL predicted proteins, 16 AtSPL proteins and 15 SlySBP proteins. The tree was generated with MEGA v6.0 software, using the maximum likelihood (ML) method, and bootstrap values were calculated with 500 replicates. Groups defined were shown on the outside of the circle [11]

**Fig. 3:**
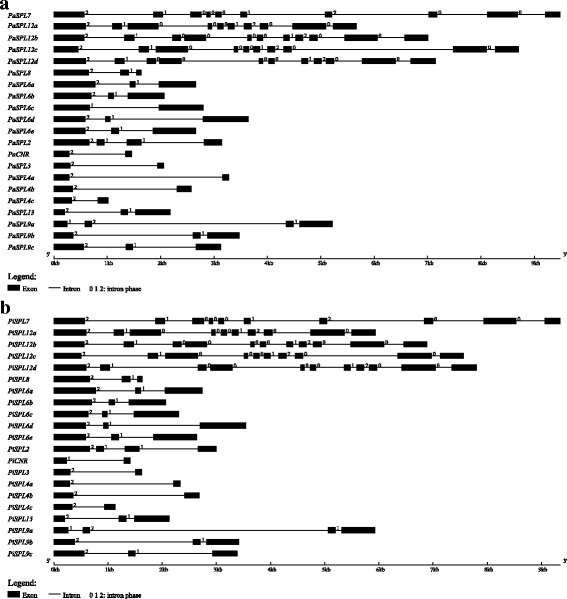
Intron-exon structures of *PaSPL* and *PiSPL* genes. **a**, Intron-exon structures of *PaSPLs.* **b**, Intron-exon structures of *PiSPLs*. 5’UTR and 3’UTR were not showed, the exons and intron were indicated by black block and gray thin line, respectively. The 0, 1 and 2 represented intron phases

To figure out the evolutionary relationship of *PhSPLs*, an unrooted phylogenetic tree was constructed using the highly conserved SBP-domains (76 aa, Fig. 1) of 21 petunia SPL proteins (PhSPLs), 16 *Arabidopsis* SPL proteins (AtSPLs) and 15 tomato SPL proteins (SlySBPs) by the maximum likelihood algorithm in MEGA 6.0, which demonstrated that 21 petunia SPL proteins were clustered into eight groups (Group I to Group VIII), and each group contained at least one member from *Arabidopsis* and tomato (Fig. 2). The Group I and Group VII contained only one SPL from petunia, Arabidopsis and tomato (PhSPL7, AtSPL7 and SlySBP7 in Group I; PhSPL13, AtSPL13 and SlySBP13 in Group VII), respectively, suggesting potential functional conservation of *SPL7* and *SPL13* genes in plants. The Group II consisted of four petunia SPLs (PhSPL12a to PhSPL12d), four Arabidopsis SPLs (AtSPL1, AtSPL12, AtSPL14 and AtSPL16), and two tomato SPLs (SlySBP12a and SlySBP12b). The Group III contained also only one SPL in petunia (PhSPL8) and Arabidopsis (AtSPL8), but two in tomato (SlySBP8a and SlySBP8b). The Group IV contained five petunia SPLs (PhSPL6a to PhSPL6e), three tomato SPLs (SlySBP6a to SlySBP6c), but only one Arabidopsis genes (AtSPL6), suggesting the fast gene duplication of SPL6 clade in petunia and even in Solanaceae family. The Group V consisted of three Arabidopsis SPLs (AtSPL2, AtSPL10 and AtSPL11), two tomato SPLs (SlySBP2 and SlySBP10) and one petunia SPL (PhSPL2)*.* PhCNR, PhSPL3, and PhSPL4a/b/c were clustered in Group VI, and PhSPL9a, PhSPL9b and PhSPL9c belong to Group VIII. The recognition of these different protein groups was reflected in their different gene length and genomic intron-exon structures (Fig. 3).

### Sequence feature and gene structure of *Petunia SPL* genes

Sequence feature analysis indicated that the length of *Petunia SPL* genes varied from 1024 bp (*PaSPL4c*) to 9487 bp (*PaSPL7*) in *P. axillaris* (Additional file 1) and 1133 bp (*PiSPL4c*) to 9350 bp (*PiSPL7*) in *P. inflata* (Additional file 2). The ORF length of *PhSPLs* varied from 285 bp (*PhCNR*) to 3027 bp (*PhSPL12d*); the size of deduced PhSPL proteins varied between 94 and 1008 amino acids (Table 1). Gene structure analysis showed that the number of exon was diverse in different groups, but was nearly consistent in the same group, such as genes in Group VI (*SPL3* and *SPL4a/b/c*) have two exons, genes in Group IV (*SPL6a* to *SPL6e*) contain 3 exons, with the exception of *PaSPL6c* that seems have lost the second exon, whereas genes in Group I (*SPL7*) and Group II (*SPL12a* to *SPL12d*) possess 10–11 exons (Fig. 3, Additional files 1 and 2). All *Petunia SPL* genes were found to have an intron at an evolutionary highly conserved position in the SBP domain, although its length varied greatly among the members from the shortest 117 bp in *PiSPL2* to the longest 4396 bp in *PiSPL9a*.

### Origin of *PhSPL* genes

**Fig. 4:**
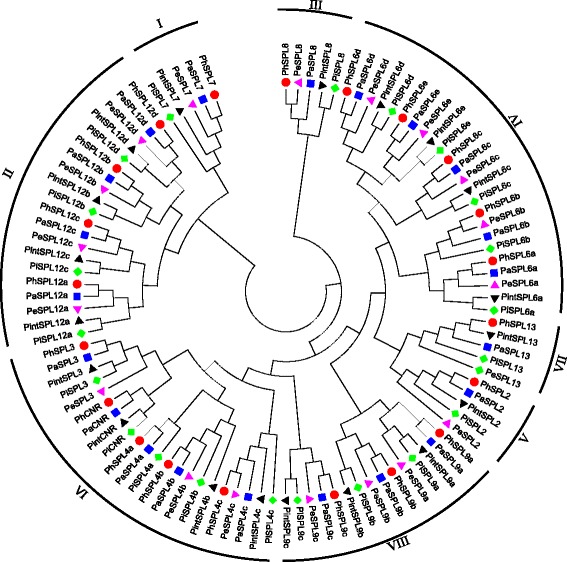
Phylogenetic tree based on the cDNA sequence alignment of *PhSPL*s, *PaSPL*s, *PiSPL*s, *PintSPL*s and *PeSPL*s. *PaSPL*, the *SPL* genes of *P. axillaris*. *PiSPL*, the *SPL* genes from *P. inflata*. *PintSPL*, the *SPL* genes from *P. intergrifolia*. *PeSPL*, the *SPL* genes from *P. exserta*. The tree was generated with MEGA v6.0 software using the Neighbor-Joining (NJ) method and 1000 bootstrap replicates

To understand the origin of *PhSPL* genes, the coding sequences of *PhSPLs* were used to query the Transcriptome Shotgun Assembly (TSA) database in NCBI of another two wild *Petunia* species, *P. integrifolia* and *P. exserta* [52]. Finally, nineteen *SPL* genes were recognized in *P. integrifolia* (designated as *PintSPLs*, Additional file 2) and *P. exserta* (designated as *PeSPLs*, Additional file 3), respectively. To elucidate whether *P. axillaris*, *P. inflata*, *P. integrifolia* or *P. exserta* is the donor of *PhSPL* genes in hybrid petunia, multiple alignments of the nucleotide sequences (CDS region) of *PhSPLs*, *PaSPLs*, *PiSPLs*, *PintSPLs* and *PeSPLs* was conducted, and then a phylogenetic tree was constructed by the Neighbor-Joining (NJ) method. The phylogenetic analysis showed that 17 of 21 *PhSPL* genes, including *PhSPL*2, *PhCNR*, *PhSPL3, PhSPL4a/b*, *PhSPL6a*, *PhSPL6c/d/e*, *PhSPL7*, *PhSPL9a/b/c*, and *PhSPL12a/b/c/d*, were clustered together with their orthologs from *P. axillaris*, and three *PhSPL*s (*PhSPL4c*, *PhSPL6b* and *PhSPL*8) were grouped together with the orthologs of *P. exserta*. Interestingly, only one *PhSPL*s (*PhSPL13*) were grouped together with the orthologs of *P. integrifolia*, and no *PhSPL* gene was clustered together with the orthologs from *P. inflata* (Fig. 4), implying that most *PhSPL* genes might be derived from *P. axillaris* or *P. exserta*.

To further confirmed the relationship of these *SPL* genes, the coding sequences of orthologs from *P. hybrida* W115, *P. axillaris*, *P. integrifolia*, *P. inflata* and *P. exserta*, respectively, were aligned and compared for each member. The results showed that the coding sequences of *PhCNR*, *PhSPL3*, *PhSPL6a*, *PhSPL6d*, *PhSPL7* and *PhSPL12a/b/c* were exactly the same as that of the orthologs from *P. axillaris*, but different at least for two nucleotides from that of the orthologs in *P. inflata*, *P. integrifolia* and *P. exserta* (Additional file 4), indicating that these genes are originated from *P. axillaris*; the coding sequences of *PhSPL4c* was identical to that of *PeSPL4c*, indicating it was most probably originated from *P. exserta. PhSPL6b* as well as *PhSPL8* showed few (1 or 2) and same number of nucleotide differences compared to the orthologs of *P. axillaris* and *P. exserta*, but with more changes (23 and 13/11 nucleotides, respectively) in comparison to *PiSPL6b* and *PiSPL8*/*PintSPL8*, and the differential 1–2 nucleotides are also different from that of *PiSPL6b* and *PiSPL8/PintSPL8*, suggesting *PhSPL6b* and *PhSPL8* were derived from *P. axillaris* or *P. exserta* (the coding sequences of *PaSPL6b* and *PeSPL6b* as well as *PaSPL8* and *PeSPL8* are identical). For the rest of *PhSPLs* except *PhSPL6c* and *PhSPL13*, also only few (1–8) nucleotides are different when compared with *PaSPLs*, while when compared with *PiSPLs*, *PintSPLs* and *PeSPLs,* especially the sequences from *PiSPLs* and *PintSPLs*, more nucleotide and amino acid changes were found (Additional file 4). Further inspection of the differential nucleotides showed that changed nucleotides in *PhSPL4a*, *PhSPL6e*, and *PhSPL12d* are also different from the genes of *P. inflata*, *P. integrifolia* and *P. exserta*, suggesting they may be derived from *P. axillaris* or originated from other relatives; in *PhSPL4b,* the varied 3 nucleotides are located in the latter part of the coding sequence that is identical to the sequence of *PeSPL4b*, indicating it may be derived from the recombination of *PaSPL4b* and *PeSPL4b*, i.e. a hybrid origin of *P. axillaris* and *P. exserta*; in *PhSPL2* and *PhSPL9a/b/c*, some of the varied nucleotides are identical to the sequences of *P. inflata*, *P. integrifolia* and/or *P. exserta* orthologs, while the others are different from the genes of all four species, suggesting these members may also have a hybrid origin between *PhSPLs* and *PeSPLs* or *PiSPLs/PintSPLs*. *PhSPL13*, however, showed fewer nucleotide changes compared to *PeSPL13* and *PintSPL13* than to *PaSPL13*, and 3 of 4 different nucleotides between *PhSPL13* and *PeSPL13* are identical to the sequences of *PintSPL13* and *PaSPL13,* while the other one is *PhSPL13*-specific, indicating it may be derived from the recombination of *PeSPL13* and *PintSPL13* or *PaSPL13*. As for *PhSPL6c*, it is strange that its coding sequence was almost identical (only one nucleotide variation) to that of *PaSPL6c* (accession number: GBRU01020485.1) from the transcriptome database of *P. axillaris* [52], but the *PaSPL6c* gene identified in the genome of *P. axillaris* N [50] showed an extended first exon (635 to 670 bp) and loss of the second exon (89 bp), so resulting in 66 nucleotide changes between *PhSPL6c* and the *PaSPL6c* identified from the genome, which was confirmed by our recent transcriptome data of *P. axillaris* N [53]. Excluding the extended and lost sequences of the genome *PaSPL6c,* the rest part of coding sequence was identical to that of *PhSPL6c*, suggesting *PhSPL6c* should also be derived from *P. axillaris.*

### Conserved domains and motifs of PhSPL proteins

The identified 21 PhSPL proteins were analyzed for conserved domains using the Conserved Domain Database in NCBI, which showed that all PhSPL proteins contained the conserved SBP domain. Multiple alignments of the SBP domains demonstrated that all SBP domain of PhSPLs except that of PhCNR contained two zinc finger motifs, Zn1 (CCCH type) and Zn2 (CCHC type), and a C-terminal nuclear localization signal (NLS), like that in other plant species (Fig. 1). The PhCNR protein terminated at the beginning of Zn2, so lacking the second zinc finger motif and NLS. Furthermore, four PhSPLs (PhSPL12a to PhSPL12d) belong to Group II contained an ANK domain with two ankyrin repeats, which mediate protein-protein interactions [54], and this is consistent with previous reports in other plants.

**Fig. 5:**
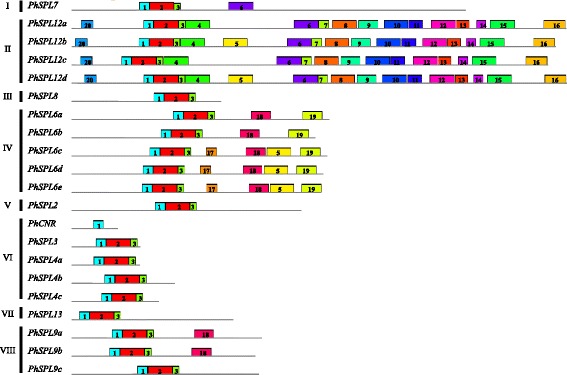
The conserved and potential motifs in PhSPL protein sequences predicted by MEME

The MEME was used to analyze conserved and potential motifs in PhSPL protein sequences (Fig. 5, Additional file 5). The number of motifs in each PhSPL varies from 1 to 17 (Fig. 5). Three motifs (motif 1, 2 and 3) constitute the SBP domain, and are conserved in all PhSPLs except for PhCNR which lack motif 2 and 3. Motif 1 is located at the first Zn finger; Motif 2 and 3 present the second Zn finger and NLS, respectively. PhSPL12a/b/c/d (Group II) are the longest group of proteins and correspondingly possess maximum number of motifs as compared to other PhSPLs. For example, motif 4, 7–16, and 20 are all exclusively present in PhSPL12a/b/c/d proteins. Motif 5 was recognized in two members of Group II (PhSPL12b and PhSPL12d) and three members of Group IV (PhSPL6c/d/e) proteins. Motif 6 was shared by all members of Group I (PhSPL7) and Group II (PhSPL12a-d) proteins. Motif 17 was only recognized in PhSPL6c, PhSPL6d and PhSPL6e. Motif 17 was found in all Group IV proteins (PhSPL6a-e) and two Group VIII proteins (PhSPL9a and PhSPL9b). Motif 19 was present specially in the Group IV members.

### *PhSPL*s targeted by miR156/157

**Fig. 6:**
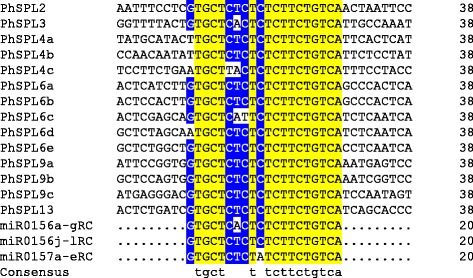
Sequence alignment of petunia miR156/157 complementary sequences with the target sites in *PhSPL* genes. The target sites are located in the coding regions with the exception of *PhSPL3*, *PhSPL4a*, *PhSPL4b* and *PhSPL4c* where they are located in the 3’-UTR. RC, reverse complementary sequence of the mature petunia miR156/157

In order to understand the miR156-mediated posttranscriptional regulation of *PhSPL* genes, we searched the CDS and 3’-UTR sequences of all *PhSPLs* for target site identification of petunia miR156 and miR157. Previously, ten *miR156* (*miR0156a-g* and *miR0156j-l*) and five *miR157* (*miR0157a-e*) genes were identified from petunia genome, which produced three kinds of mature miRNA sequences (miR0156a-g, miR0156j-l and miR0157a-e) [50]. A comparison of the mature PhmiR156/157 sequences to *PhSPLs* transcript sequences showed that 14 out of 21 *PhSPL* genes contained sequences complementary to the PhmiR156 or PhmiR157 mature sequences with one to two mismatches as maximum (Fig. 6), which suggests that PhmiR156/157 may specifically target these gene in petunia. These putative miR156/157 response elements (MREs) of *PhSPL* genes were located downstream of the SBP-box in the coding region of genes in groups IV (*PhSPL6a-e*), V (*PhSPL2*), VII (*PhSPL13*) and VIII (*PhSPL9a/b/c*), while in the case of members in Group VI (*PhSPL3* and *PhSPL4a/b/c*), it was present in the 3’-UTR regions.

### Expression patterns of *PhSPL* genes

**Fig. 7:**
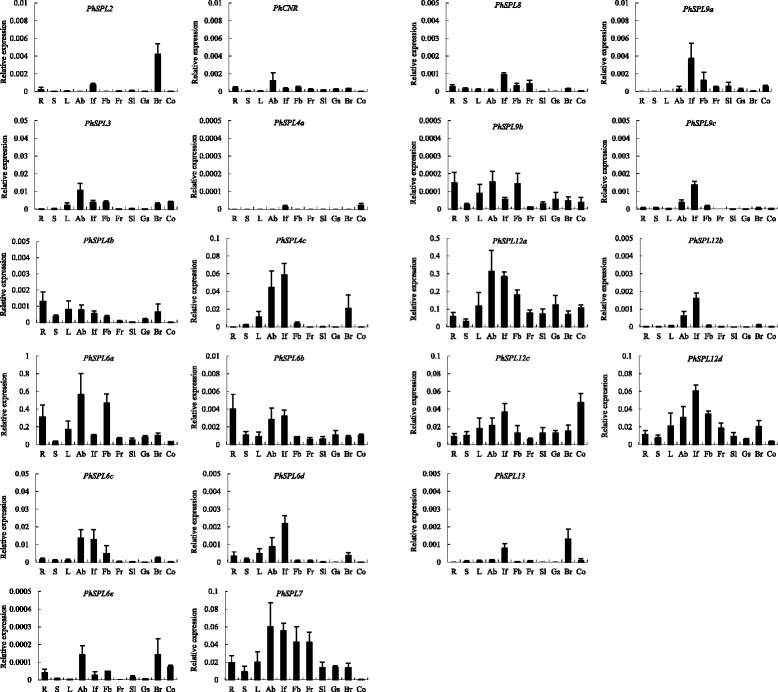
Expression patterns of *PhSPL* genes in petunia. R, roots (7d after flowering); S, stems (7d after flowering); L, leaves (7d after flowering); Ab, axillary buds (7d after flowering); If, inflorescences; Fb, flower buds (0.5 cm); Fr, fruits (7d after pollination); Sl, young seedlings (4 euphylla seedling); Gs, germinal seeds (3 d after sowing); Br, bracts; and Co, cotyledons (7 d after sowing). The level of expression was normalized to petunia *EF1α* gene. Error bars represent SE for three replicates

Transcription activity of *PhSPL* genes was investigated in various tissues using qPCR with gene-specific primers (Additional file 6). According to their expression patterns (Fig. 7, Additional file 7), *PhSPL* genes can be roughly classified into several groups. The first group was ubiquitiously expressed at relatively high levels in all tested tissues, such as *PhSPL6a*, *PhSPL6b*, *PhSPL7*, *PhSPL12a*, *PhSPL12c* and *PhSPL12d*. *PhSPL6a* and *PhSPL12a* showed the highest expression levels in most tissues among all *PhSPL* genes, with *PhSPL6a* expressed higher in axillary buds, flower buds, and roots than in other tissues, *PhSPL12a* expressed higher in axillary buds, inflorescences and flower buds than in other tissues (Fig. 7). *PhSPL6b* was expressed at significantly lower level (about 100 folds less) than *PhSPL6a*, with more expression in roots, inflorescences, and axillary buds than in other tissues. *PhSPL7* showed the expression level of higher (about 10 folds more) than *PhSPL6b*, but lower (about 10 folds less) than *PhSPL6a*, with higher expression in axillary buds, inflorescences, flower buds and young fruits. *PhSPL12c* and *PhSPL12d* displayed comparable expression levels to the *PhSPL7*, but they have different expression pattern, for instance, *PhSPL12c* was expressed at the highest level in cotyledons, followed by inflorescences, while *PhSPL12c* showed highest expression in inflorescences with the lowest expression level in cotyledons. The second group had no expression or was expressed at very low levels in all tissues, such as *PhSPL4a, PhSPL6e* and *PhSPL9b*. No expression was detected for *PhSPL4a* in most tissues but very weak expression in inflorescences and cotyledons. *PhSPL6e* and *PhSPL9b* showed the expression levels less than millesimal of the first group genes, with *PhSPL6e* mainly expressed in axillary buds and bracts, and *PhSPL9b* mainly in roots, axillary buds and flower buds. The third group was expressed specially in some tissues, including *PhSPL2, PhCNR*, *PhSPL3, PhSPL4b*, *PhSPL4c*, *PhSPL6c*, *PhSPL6d*, *PhSPL8*, *PhSPL9a*, *PhSPL9c, PhSPL12b*, and *PhSPL13*. *PhSPL2* and *PhSPL13* were expressed in bracts and inflorescences. *PhCNR* and *PhSPL3* were expressed mainly in axillary buds followed by inflorescences and flower buds, but the expression levels of *PhCNR* were significantly lower than that of *PhSPL3*. *PhSPL4b* and *PhSPL4c* was expressed in most tissues including stems, leaves, axillary buds, inflorescences, flower buds and bracts, but almost no transcripts were detected in fruits, young seedlings and cotyledons. *PhSPL6c* and *PhSPL6d* were expressed mainly in axillary buds and inflorescences. *PhSPL8* was expressed mainly in inflorescences, flower buds and fruits. *PhSPL9a*, *PhSPL9c* and *PhSPL12b* showed similar expression patterns, with the highest expression level in inflorescences followed by axillary buds or flower buds.

### Overexpression of *PhSPL9a* and *PhSPL9b* in *Arabidopsis*

To characterized the functions of *PhSPL9a* and *PhSPL9b*, two co-orthologs of *AtSPL9* and *AtSPL15*, we constructed expression vectors *35S:PhSPL9a* and *35S:PhSPL9b,* then introduced into *Arabidopsis*. Fifty-two and thirty-five independent T_1_ transgenic lines were achieved for *35S:PhSPL9a* and *35S:PhSPL9b*, respectively. Twenty-eight and twenty-one of T_1_ transgenic lines from *35S:PhSPL9a* and *35S:PhSPL9b*, respectively, showed earlier flowering than the empty pCAMBIA2300 plasmid transformed control plants (CK). According to the phenotypic alterations, for each gene construction three T_1_ transgenic lines whose progenies showed a 3:1 segregation ratio for kanamycin resistance, which may indicate a single-copy insertion of transgenes, were chosen for further experiment of. 32 T_2_ transgenic plants for each line were used to investigate the flowering time and floral phenotypes.

**Fig. 8:**
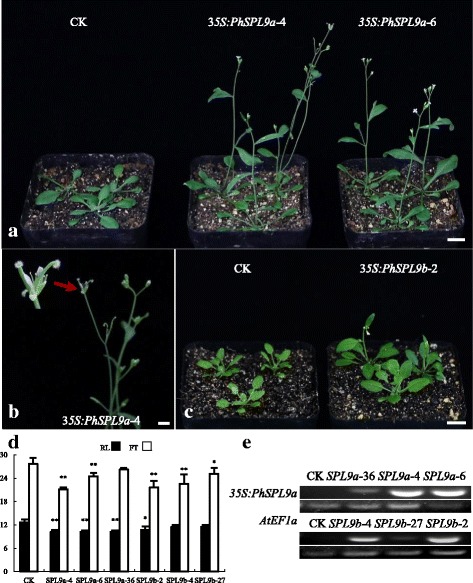
Phenotypic analyses of transgenic *Arabidopsis* plants ectopically expressing *PhSPL9a* and *PhSPL9b*. gene. **a** The control (CK, left) and *35S:PhSPL9a* transgenic plants (right) showing early flowering. **b** *35S:PhSPL9a* transgenic plants showing determinate inflorescence with a terminal polygynoecial flower (close-up). **c** CK (left) and *35S:PhSPL9b* transgenic plants (right) showing early flowering. **d** The numbers of rosette leaves (RL) and flowering time (FT) in CK, *35S:PhSPL9a* and *35S:PhSPL9b* transgenic lines; asterisks indicate statistically significant differences (* *P* < 0.05, ** *P* < 0.01) from the CK plants. **e** RT-PCR analysis of transgenes in CK and transgenic *Arabidopsis* lines. Bars: 10 mm (**a**, **c**), 1 mm (**b**)

Under long day conditions, the control plants initiated flowering when average 12.7 ± 0.8 rosette leaves and 2.3 ± 0.5 cauline leaves were produced (Additional file 8). In contrast, transgenic lines that exhibit severe phenotype such as *PhSPL9a*-4 bolted obviously earlier than CK, with significantly (*P* < 0.01) decreased number of rosette leaves and usually determinate inflorescence that terminated with a polygynoecial flower (Fig. 8b). Most transgenic lines of *35S:PhSPL9a* and *35S:PhSPL9b* showed moderate phenotypes, which bolted and flowered after producing nine to eleven rosette leaves (Fig. 8a, c; Additional file 8) under the same condition. However, there was no significant difference in the number of cauline leaf between the transgenic plants and CK.

RT-PCR analysis indicated that the phenotypic variations are related in a large extent to the expression levels of the transgenes, namely higher expression levels tend to result in more severe phenotypic changes such as earlier bolting and flowering time, and vice versa (Fig. 8d, e; Additional file 8).

## Discussion

### Evolution of *SPL* genes in *Petunia*

The SBP-box proteins (SPLs) constitute a family of transcription factors that play diverse roles in plant growth and development. Phylogenetic evidence indicated that SBP-box genes had underwent several ancient and more recent duplication events, resulted in the formation and retention of multiple *SPL* paralogs and clades [8]. Salinas et al. [11] reported eight (I-VIII) major *SPL* clades in plants based on a phylogenetic tree of 105 *SPL* protein sequences from seven species using the neighbor-joining (NJ) algorithm, at least four of which predate the diversification of land plants. A latter phylogenetic analysis with a slightly different dataset of *SPL* genes using the maximum likelihood (ML) method supported the eight major clades, and suggested a possible ninth clade containing *OsSPL14/17* from rice and *ZmSBP6/8* from maize [8]. Recently, phylogenetic reconstruction with 104 full length SBP-box protein sequences from five species including moss, *Arabidopsis*, poplar, rice and maize demonstrated that the SBP-box genes form two lineages (group I and II) through an early duplication event, and the latter is further divided into two distinct subgroups (subgroup II-1 and II-2) caused by several rounds of duplication [55].

In this work, the *SPL* genes were identified from four *Petunia* species at genome or transcriptome scale, followed by cloning and sequencing of the orthologs from a hybrid petunia line W115. As a result, 21 putative *SPL* genes were identified in the genome of *P. axillaris* (*PaSPLs*) and *P. inflata* (*PiSPLs*), respectively, 19 *SPL* genes were recognized from the transcriptome of *P. integrifolia* (*PintSPLs*) and *P. exserta* (*PeSPLs*), respectively, and 21 *SPL* genes were isolated from petunia line W115 (*PhSPLs*), a number similar to that found in *Nicotiana tomentosiformis* (Additional file 9), grape [12], rice [3], and *Ziziphus jujuba* [56]. However, in contrast to several other species in Solanaceae family, including tomato [11], pepper [18], and potato [57], *Petunia* appears to contain a larger number of *SPL* genes (21 vs. 15), suggesting possible more duplication events of *SPL* genes in *Petunia* genome or faster loss of some members in other Solanaceae species. For instance, there are two orthologs in petunia corresponding to one gene in tomato for several members, such as *PhSPL4a* and *PhSPL4b* vs. *SlySBP4*, *PhSPL6a* and *PhSPL6b* vs. *SlySBP6b*, *PhSPL12a* and *PhSPL12c* vs. *SlySBP12a*, *PhSPL12b* and *PhSPL12d* vs. *SlySBP12b*, and *PhSPL9a* and *PhSPL9b* vs. *SlySBP15* (Fig. 6). The *PhSPL12b* and *PhSPL12d* gene pair was evidently produced by a more recent additional duplication in *Petunia*, but for other members it also could be resulted from the loss of duplicated genes in tomato. In addition, tomato has no counterparts of *PhSPL4c* and *PhSPL9c*, suggesting they may also had lost. The duplicated genes are usually followed by two kinds of destiny: loss of function by pseudogenization, or evolution of function via subfunctionalization or neofunctionalization. *Petunia* contains more *SPL* genes than the other Solanaceae species suggests that the *SPLs* in *Petunia* may undergo fast functional evolution. Similarly, there are much more Group IV *SPL* members in *Petunia* than in *Arabidopsis*, five vs. one, suggesting these duplicated genes must undergo fast subfunctionalization or neofunctionalization, and may play redundant but divergent functions. Phylogenetic analysis indicated that 21 petunia *SPL* genes can be divided into eight groups (Group I-VIII), which is consistent with the results of *SPLs* from *Arabidopsis* [8] and tomato [11]. The Group I is corresponding to the first lineage (group I) described by Zhang et al. [55], and contains only one member (*PhSPL7*); the Group II is corresponding to the subgroup II-1 in the second lineage described by Zhang et al. [55], and contains 4 members (*PhSPL12a-d*). This phylogenetic relationship is in accordance with the result described by Zhang et al. [55], but different from that reported by Preston and Hileman [8], maybe due to different algorithms being used. Within each group, the exon-intron structures are almost constant, but it varied among different groups, such as the Group I and II genes are evidently longer and contain more exons compared to the other groups (10–11 vs. 2–4), which is consistent with the situation in most species, including monocots rice and maize [8], suggesting the *SPL* gene structures are relatively conserved during the evolution.

All PhSPLs contained highly conserved SBP domain with 76 amino acid residues except for PhCNR that possess only the front part of the SBP domain (Fig. 1) and lack the motif 2 and 3 (Fig. 5) due to a nonsense mutation at the middle of SBP domain, i.e. *PhCNR* gene contains a premature stop codon and encodes a truncated protein with only 94 amino acids (Table 1). The same mutation was found in *PaCNR* of *P. axillaris* (Additional file 1) but not in *PiCNR* of *P. inflata*, however, a 55-bp deletion resulting in frame shift and premature translation termination was found in *PiCNR* (Additional file 2), suggesting that the *CNR* in *Petunia* might be pseudogenization, which was supported further by the fact that no *CNR* ortholog was identified in *P. exserta* transcriptome (Additional file 3), but it was indeed expressed in *P. axillaris, P. inflata,* and W115. Similar motifs and their positions were revealed in the same group of PhSPLs and comparable with the results in other plant species, especially for the Group I and II genes, which supports the results reported by Zhang et al. [55] that group I and subgroup II-1 genes are constrained by stronger purifying selection and evolved at a lower substitution rate than II-2 genes. The erratic gene is *SPL9c*, which was phylogenetically closer to the genes of Group IV (Fig. 2), but has more similar gene length and intron-exon structure to members in Group VIII (Additional files 1 and 2), so we designated it as the genes in Group VIII. However, unlike the other two members (SPL9a and SPL9b) of Group VIII, SPL9c lack the motif 18, which is similar to the members in Group IV (Fig. 5).

Multiple alignments of the coding sequences of *PhSPL* genes with the orthologs from *P. axillaris*, *P. inflata*, *P. integrifolia*, and *P. exserta* demonstrated that most *PhSPL* genes were derived from *P. axillaris,* which is in consistent with the fact that *P. hybrida* ‘Mitchell’ is a backcross hybrid of *P. axillaris* × (*P. axillaris* × *P. hybrida* ‘Rose of Heaven’). Sequence alignment analysis in our study suggested that several *PhSPL* genes including *PhSPL2*, *PhSPL4b*, *PhSPL9a/b/c* and *PhSPL13* may have a hybrid origin from *P. axillaris* and *P. integrifolia/P. inflata* or some other wild species such as *P. exserta*.

It has been reported that most SBP-box family of genes are targeted by miR156/157 in a number of plant species, such as 10 of 16 *Arabidopsis SPL* genes are the targets of miR156/157 [3, 23], 11 of 19 *OsSPL* genes are predicted to be targeted by OsmiR156 in rice [3, 23], and 10 of 15 *SPL* genes were found to be targets of miR156 in tomato [11]. A target search of PhmiR156/157 against the coding regions and 3’ UTRs of petunia *SPLs* indicated that 14 out of 21 (two-thirds) *PhSPL* genes were potential targets of PhmiR156/157. All the putative PhmiR156-targeted *PhSPL* genes were clustered into groups IV to VIII, and miR156/157-targeting sites in 10 *PhSPLs* belonging to groups IV, V, VII and VIII locate in the coding region, while the target sites for other 4 *PhSPLs* belonging to group VI locate in the 3’ UTRs (Fig. 6), which is consistent with the results from other plants [58], suggesting miR156/157-mediated post-transcriptional regulation of *SPLs* is a highly conserved mechanism in plants. In animals, miRNAs are prone to act by repressing translation and their target sites are mostly found in 3’ UTRs, however, plant miRNAs were believed to exert their function mainly by mediating target cleavage, so the target sites are often located in ORFs that may facilitate the destruction of target mRNA after cleavage. Besides cleavage, accumulating genetic evidence indicates that plant miRNAs also can mediate translation repression, in which situation the target sites could be located in 3’ UTRs or ORFs [59]. Petunia group IV, V, VII and VIII *SPL* genes contain their miR156/157-binding sites in the coding region suggests they may be regulated by miRNA-mediated cleavage, while the group VI genes with the target sites in 3’ UTRs should be controlled by translation repression.

### Expression patterns and potential functions of *PhSPL* genes

At present, there was few functional data of *SPL* genes available in petunia. Usually, the gene functions are expected to correlate in a large extent with their expression patterns. In this study, the expression of *PhSPL* genes in various tissues were investigated by qPCR analysis, which indicated *PhSPLs* exhibited distinct expression patterns in terms of specificity and expression level (Fig. 7). Similar to the results from many other species, where *SPL* genes exhibited higher expression levels in the shoot apices (apical buds), inflorescences (panicles), and flower buds [11, 13, 60], most *PhSPL*s expressed predominantly in the axillary buds, inflorescences, and/or flower buds (Fig. 7), suggesting they might be involved in the development of these organs in petunia. There is no obvious correlation between gene structures and expression patterns, although some genes within the same group demonstrated similar expression profiles.

*SPL* Group I and II genes are characterized by their large size and lack of miR156 and miR157-binding sites. Previous studies demonstrated that these two groups of *SPL* genes were almost ubiquitously expressed across different plant organs [8, 55]. For instance, *SlySBP7*, the Group I gene in tomato, is expressed in seedlings, roots, stems, leaves, vegetative shoot apices, inflorescences, flowers, and fruits [11]; the four *Arabidopsis* Group II genes, *AtSPL1*, *AtSPL12*, *AtSPL14*, and *AtSPL16*, are expressed wildly in seedlings, roots, rosette and cauline leaves, shoot apical meristem, flowers, and fruits [3, 8]. Petunia Group I (*PhSPL7*) and Group II (*PhSPL12a/b/c/d*) genes also showed ubiquitous expression in all tested tissues, although they were expressed most strongly in different tissues, and *PhSPL12c* exhibited much lower expression levels than other genes (Fig. 7). Currently, the only functional analysis of Group I genes is limited to *Arabidopsis SPL7* (*AtSPL7*), which was characterized to play a pivotal role in the regulation of copper homeostasis [32]. *PhSPL7* may have similar functions to *AtSPL7* based on their conserved gene structures and expression patterns. Functional data for Group II genes exist for two members, *PpSBP2* and *AtSPL14*. Like *AtSPL7*, *PpSBP2* is involved in the regulation of copper homeostasis in *Phycomitrella patens* [61], while *AtSPL14* is involved in response to the fungal toxin fumonisin B1 (FB1) and may also function to delay the juvenile to adult transition [38]. Functional diversification in Group II make the function prediction of *PhSPL12a/b/c/d* genes unreliable, while their expression patterns suggest that they should play redundant roles in regulating the development of axillary buds, inflorescences and/or flowers, with *PhSPL12c* may also in cotyledon development. It might also be of interest to investigate if *PhSPL12a* to *PhSPL12d* are associated with programmed cell death in response to the FB1 in petunia.

Group III *SPL* genes also lack the miR156/157-binding site, although short relative to Group I and II genes. The first Group III gene to be functionally characterized was *AtSPL8* in *Arabidopsis,* which is a tissue-dependent regulator of GA response and regulate early anther development and gynoecium differential patterning, trichome formation on sepals, stamen filament elongation, and root growth [34–37]. High expression of the *AtSPL8* orthologs *SlySBP8a* and *SlySBP8b* in inflorescences, carpels, and young fruits but very low in roots, seedlings, and stamens of tomato suggests at least partial conservation of function in gynoecium differentiation [11]. In maize and rice, *AtSPL8* ortholog (*LG1* and *OsLG1*, respectively) is involved in development of the ligule and auricule [42, 43], and controls the branch angle of tassel and panicle, respectively [44–46]. *PhSPL8* was expressed more highly in inflorescences, flower buds, and young fruits versus other tissues, suggesting it may play partial conserved roles in inflorescence, anther and gynoecium development.

Group IV contains only one *SPL* gene (*AtSPL6*) in *Arabidopsis*, but three genes (*SlySBP6a/b/c*) in tomato, five genes (*PhSPL6a/b/c/d/e*) in petunia (Fig. 2), five genes (*PpSBP3/6/6b/13/14*) in *Phycomitrella*, and no orthologs in the fully sequenced genomes of rice and other grasses [8], suggesting a loss of this gene lineage at least in some monocots and differential duplication and expansion in different species. *AtSPL6* and its ortholog *NbSPL6* of *Nicotiana benthamiana* play a conserved role in the TIR-NB-LRR receptor-mediated plant innate immunity [31], while the function of *PpSBP3* was characterized to repress reproduction development [62]. In petunia, expression of the five Group IV *SPL* genes suggests divergence of function. Similar to *AtSPL6* and *SlySBP6a* [10, 11], *PhSPL6a* and *PhSPL6b* were expressed constitutively across the plant, but *PhSPL6a* expressed predominantly in axillary buds, flower buds, and roots while *PhSPL6b* expressed mainly in roots, inflorescences, and axillary buds. By contrast, *PhSPL6c* and *PhSPL6d* expression was somewhat tissue-specific and confined primarily to the axillary buds and inflorescences; *PhSPL6e* showed very low expression and mainly in axillary buds, bracts and cotyledons (Fig. 7). The additional duplications of *SPL6* in petunia also suggest functional redundancy, which need to be characterized yet.

Group V contains three *SPL* genes (*AtSPL2*, *AtSPL10*, and *AtSPL11*) in *Arabidopsis*, while only one member (*PhSPL2*) exists in petunia. Recent studies demonstrated that *AtSPL2/10/11* have overlapping functions in promoting vegetative phase change and flowering [9]. *AtSPL2* might also control floral organ development and plant fertility by directly regulating *ASYMMETRIC LEAVES 2* (*AS2*) expression [28]. It is interesting that *PhSPL2* was expressed mainly in bracts and inflorescences, which is different from the constitutive expression of *AtSPL2/10/11* and tomato orthologs *SlySBP2* and *SlySBP10* [11, 63]. The special expression pattern of *PhSPL2* suggests a distinct function from *Arabidopsis* genes, which remains to be revealed.

*AtSPL3*/*4/5* are Group VI *SPL* genes in *Arabidopsis*, which were previously considered to redundantly promote vegetative phase change and flowering based on the overexpression data [29]. Recent detailed analysis of *spl* mutants, however, revealed that *AtSPL3/4/5* play a major role in floral meristem identity transition rather than in vegetative morphogenesis or flowering induction [9]. Similar to *Arabidopsis*, the single *AtSPL3/4/5* ortholog *AmSBP1* in snapdragon (*Antirrhinum majus*) is involved in initiating flower development after the switch to inflorescence development; silencing of *AmSBP1* also causes an increase in vegetative branching under long days [64]. By contrast, the tomato *AtSPL3* ortholog *CNR* is required for fruit ripening [47]. In petunia, there are five Group VI *SPL* genes, *PhSPL3*, *PhSPL4a*, *PhSPL4b*, *PhSPL4c*, and *PhCNR*, with significantly different expression patterns and levels (Fig. 7). A recent study demonstrated that *PhSBP1* (*PhSPL3*) and *PhSBP2* (*PhSPL4c*) differentially promote discrete stages of the reproductive transition, and *PhSBP1,* possibly also *PhCNR,* accelerates leaf initiation rate [49]. This is consistent with the relatively high expression of *PhSPL3* and *PhSPL4c* in axillary buds, inflorescences, and late stage of shoot apices (Fig. 7) [49]. *PhCNR* also expressed mainly in axillary buds, however, our sequence analysis demonstrated that *PhCNR*, as well as *PaCNR* and *PiCNR*, is likely to be pseudogene due to nucleotide change or deletion mutation. Compared with *PhSPL3* and *PhSPL4c*, *PhSPL4a* and *PhSPL4b* showed much lower expression, especially *PhSPL4a* that only expressed very faintly in inflorescence and cotyledon (Fig. 7). The differential expression was also found in tomato Group VI *SPL* genes, *SlySBP3*, *SlySBP4*, and *CNR* [11], suggesting divergent and elusive evolution of functions in this group.

Expression and functional data demonstrated that Group VII *SPL* genes were involved in various aspects of above ground plant development. In *Arabidopsis*, the single *SPL* Group VII gene *AtSPL13* has been implicated in the regulation of the post-germinative switch from the cotyledon stage to the vegetative-leaf stage [65]; it also plays overlapping roles with *SPL2/10/11* and *SPL9/15* in promoting vegetative and reproductive phase change [9]. In contrast, the *AtSPL13* ortholog in maize and rice, *TGA1* and *OsSPL16*, is involved in ear glume development [39] and controls grain size, shape and quality, respectively [40, 41], suggesting divergent functional evolution of the Group VII *SPL* genes. Different from *AtSPL13* that is expressed highly in the hypocotyl, shoot apical meristem, leaf primordia, and developing inflorescence [9, 65], *PhSPL13* expressed mainly in inflorescence and bract in petunia (Fig. 7), suggesting it might have distinct functions from *AtSPL13*.

Similar to *AtSPL2/10/11*, *AtSPL9* and *AtSPL15* belonging to Group VIII also contribute to both the vegetative and reproductive phase change, and play more important role than *SPL2/10/11* [9]. *AtSPL9* was also suggested to be involved in petal trichome initiation, anthocyanin pigment accumulation, and sesquiterpene biosynthesis [66–68]. The genome-wide analysis indicated that petunia contains three Group VIII *SPL* genes, *PhSPL9a*, *PhSPL9b*, and *PhSPL9c.* The expression of *PhSPL9a* and *PhSPL9c* was mainly in the inflorescences, while *PhSPL9b* was expressed constitutively in all tissues, although with relatively low expression levels (Fig. 7). Overexpression of *PhSPL9a* or *PhSPL9b* in Arabidopsis resulted in early flowering with significantly reduced number of rosette leaves, suggesting *PhSPL9a* and *PhSPL9b* may have conserved functions in promoting the vegetative-to-reproductive phase transition. However, differential expression pattern between *PhSPL9a* and *PhSPL9b* implied their distinct functions in petunia, which awaits to be characterized in the future.

## Conclusion

We identified 21 putative *SPL* genes within *P. axillaris* and *P. inflata* genome, respectively, which was confirmed by cloning from *P. hybrida* W115. The analyses of gene structure, expression pattern, and potential functions of petunia *SPLs* support that the plant SBP-box genes are conserved gene family, but underwent differential expression and functional evolution within different groups and species. The present work provides an important foundation for the future elucidation of the biological functions of petunia *SPL* genes.

## Methods

### Plant materials and bacterial strains

The plant materials used in this study included *Petunia hybrida* line W115 (Mitchel diploid) and Col-0 ecotype of *Arabidopsis thaliana*. They were grown under long-day conditions (16 h light/8 h dark) with 75% humidity in a growth chamber at 21–22 °C.

*Escherichia coli* strain DH5α was used as host in cloning of the coding sequences of *PhSPL* genes. *Agrobacterium tumefaciens* strain GV3101 was used for transformation of *Arabidopsis.*

### Identification and cloning of *SPL* genes in *Petunia*

Petunia SBP-box genes were identified from *P. axillaris* N and *P. inflata* S6 genome databases in Sol Genomics Network (https://solgenomics.net/) [50] by BLAST homology search using the nucleotide and protein sequences of 16 Arabidopsis *SPL* genes [10], 15 tomato SBP-box genes [11], and 22 *Nicotiana tomentosiformis SPL* genes (Additional file 9), respectively. The transcriptome databases of *Petunia* species including *P. axillaris*, *P. integrifolia*, *P. exserta* and *P. inflata* in the NCBI [52, 69], and *P. hybrida* ‘Mitchell’ in Sol Genomics Network [70] were searched for the transcripts of *Petunia* SBP-box genes by nucleotide BLAST with identified *Petunia SPL* genes.

To confirm the results, gene-specific primers (Additional file 6) were designed to amplify the complete coding sequences of *PhSPL* genes from *P. hybrida* line W115. Total RNA was extracted from mix samples of seedlings, leaves, inflorescences and flowers with RNA pure Total RNA Kit (Aidlab, China). 2 μg of RNA was used for cDNA synthesis (Takara, Japan), the resulting cDNA was diluted 1:10, and then 2 μl was used in PCR with 2 × High-Fidelity Master Mix DNA Polymerase (Tsingke, China). The PCR conditions were 94 °C for 4 min, followed by 32–35 cycles of 94 °C for 30 s, 55–60 °C for 30 s, 72 °C for 1–3 min, and a final extension of 72 °C for 10 min. PCR products were purified from agarose gel with Axyprep DNA Gel Extraction Kit (Axygen, USA), then cloned into pMD18-T (Takara, Japan) and transformed into *E. coli* DH5α, and 4–5 positive clones for each gene were selected randomly for sequencing analysis (Augct, China).

### Multiple sequence alignments and phylogenetic analysis

The coding sequences of the orthologous *SPL* genes in *P. axillaris*, *P. inflata* and *P. exserta* were deduced from the genomic DNA sequences and/or the cDNA sequences from transcriptome databases by BLAST search and alignment with the confirmed coding sequences of *PhSPL* genes. These nucleotide sequences were first aligned with the MUSCLE program implemented in MEGA 6.0 [71]. Phylogenetic tree of the nucleotide sequences of *PhSPL* genes and their orthologous genes in *P. axillaris*, *P. inflata* and *P. exserta* was constructed using MEGA 6.0 with the Neighbor-Joining (NJ) method and 1000 bootstrap replicates.

The SPL protein sequences of *Arabidopsis* and tomato were downloaded from the website of TAIR (http://www.arabidopsis.org/) and the tomato genome database in Sol Genomics Network, respectively. Multiple alignments of the conserved SBP domains (76 aa) of protein sequences encoded by *SPL* genes of petunia, *Arabidopsis* and tomato were conducted by the ClustalW program of MEGA 6.0. Phylogenetic relationship of SBP-box genes was estimated using the maximum likelihood (ML) method in MEGA 6.0, and bootstrap values were calculated with 500 replicates.

### Analyses of the gene structure and conserved motifs in *PhSPL* genes

The Gene Structure Display Server 2.0 (http://gsds.cbi.pku.edu.cn/) was used to predict the exon-intron structure and locations of *PhSPL* genes in the downloaded genomic sequences [72]. Conserved domains of PhSPL proteins were identified using the Conserved Domain Database (CDD) in NCBI with default parameters [73]. The MEME (Multiple Expectation Maximization for Motif Elicitation) 4.12.0 (http://meme-suite.org/tools/meme) was used to predict both conserved and potential motifs of the putative PhSPL protein sequences using the parameter settings: the minimum motif width = 6, the maximum motif width = 50, and the maximum number of motifs = 20 [74].

### Prediction of miR156/157-targeted *PhSPLs*

*PhSPLs* potentially targeted by miR156/157 were determined with petunia miR156/157 sequences [50] against the coding regions and 3’-UTRs of all PhSPLs for complementary sequences of PhmiR156/157 on the psRNATarget server (http://plantgrn.noble.org/psRNATarget/) with default parameters [75].

### Expression pattern analyses of *PhSPL* genes by real-time quantitative RT-PCR

Different vegetative and reproductive tissues were harvested from petunia line W115, represented by germinating seeds (3d after sowing), cotyledons (7d after sowing), young seedlings (four euphylla stage), roots, stems, leaves and axillary buds of adult plants (after flowering), bracts, inflorescences, flower buds (0.5 cm), and young fruits (7d after pollination). All samples were frozen in liquid nitrogen immediately after collection and stored at − 80 °C until used for qPCR analysis.

Total RNA was extracted with RNA pure Total RNA Kit (Aidlab, China). First-strand cDNA synthesis was carried out with 2 μg of total RNA for each sample using the PrimeScript™ RT reagent Kit with gDNA Eraser (Takara, Japan). Real-time quantitative RT-PCR (qPCR) was performed with the SYBR Premix Ex Taq (Takara, Japan) and the ABI7500 Real-Time System (Applied Biosystems, USA) to reveal the expression level of *PhSPL* genes in various tissues. qPCR products were amplified with 1 μl template of 20-fold diluted RT reaction mixture, 5 μl 2 × SYBR Green Master Mix, 0.2 μl forward and reverse primers (10 μmol/μl), and water to a final volume of 10 μl. The transcript levels of *PhEF1α* was determined for reference [76]. qPCR was performed with three biological replicates for each sample and the mean values ± SD (standard deviation) were calculated. Data was analyzed using the 2^−ΔΔCT^ method [77]. All the primers were designed by Primer-BLAST online (http://www.ncbi.nlm.nih.gov/tools/primer-blast/) to avoid conserved regions and listed in Additional file 6. Product size was 120–300 bp. Primer specificity was tested via PCR using recombinant plasmid DNA containing purpose fragment.

### Vector construction and plant transformation

The full-length cDNA sequences of the *PhSPL9a* and *PhSPL9b* genes were amplified and cloned into pMD-18 T vector (Takara, Japan). Sequence accuracy and insertion direction were confirmed by sequencing. Digestion of these vectors was conducted using SalI and BamHI (Takara, Japan) restriction endonuclease enzymes, and the digested products were ligated into the appropriate sites of pCAMBIA2300 vector containing the *CaMV35S* promoter, to create the constructs *35S:PhSPL9a* and 35S*:PhSPL9b*. All the constructed plasmids were confirmed by PCR and restriction digestions. *Agrobacterium tumefaciens* GV3101 containing the respective expression vectors was used for *Arabidopsis* (Col-0) transformation via the floral dip method. The transformed seeds were surface-sterilized with 8% (*v*/v) sodium hypochlorite solution for 4 min followed by 95% (v/v) ethanol for 2 min, then rinsed 3 times in sterile water and screened on half-strength Murashige and Skoog (1/2MS) medium supplemented with 50 mg/L kanamycin sulfate (Biosharp, China) and 50 mg/L cefotaxime sodium salt (Biosharp, China). The plants that survived were transplanted into soil and grown under long-day conditions (16 h light/8 h dark) at 22 °C to obtain self-pollinated seeds.

### Phenotype and transgene expression analysis

Following the segregation tests, 32 kanamycin-resistant transgenic plants of the T_2_ generation lines which fitted a segregation ratio of 3:1 were chosen to record flowering time and floral phenotype. Seedlings were grown in a growth incubator at 22 °C under a long day (LD) conditions (16/8 h, light/dark). Flowering time and the number of rosette leaves were measured when the plants bore a 1 cm long inflorescence.

RT-PCR was performed to analysis the expression of the transgene in *Arabidopsis*, which were carried out on 14-d-old seedlings. Total RNA was isolated and then reverse-transcribed from the control plants (Col-0 *Arabidopsis* transformed with the empty pCAMBIA2300 plasmid) and T_1_ transgenic lines with the same reagent kit described above. *Arabidopsis EF1α* gene was used as the reference gene to normalize small differences in template amounts. Primers used for the detection of *PhSPL9a* and *PhSPL9b* expression levels in the transgenic plants were listed in Additional file 6.

### Statistical analysis

Statistical differences were analyzed using ANOVA (analysis of variance) in IBM SPSS Statistics 22 based on Duncan’s multiple range test (*P* < 0.05).

## Additional files

Additional file 1: Orthologous *SPL* genes in *P. axillaris* N genome. ^a^Sequence ID corresponds to annotations provided by https://solgenomics.net/organism/Petunia_axillaris/genome (v1.6.2) [50]. ^b^ The transcripts were identified by nucleotide BLAST search of the TSA (Transcriptome Shotgun Assembly) database of *Petunia axillaris* in the NCBI and confirmed by alignment with AlignX program in Vector NTI Advance v11.5.2 [57]. (DOCX 18 kb)

Additional file 2: Orthologous *SPL* genes in *P. inflata* S6 genome and *P. intergifolia* transcriptome. ^a^ Sequence ID corresponds to annotations provided by https://solgenomics.net/organism/Petunia_axillaris/genome (v1.0.1) [50] ^b^ The transcripts were identified by nucleotide BLAST search of the TSA (Transcriptome Shotgun Assembly) database of *Petunia integrifolia* (GBRV) and *P. integrifolia* Subs*p. inflata* (GBDS) in the NCBI and confirmed by alignment with AlignX program in Vector NTI Advance v11.5.2 [57]. ‘/’ indicates no orthologous transcript was found. (DOCX 22 kb)

Additional file 3: Orthologous *SPL* genes in the transcriptome database of *P. exserta *[51]. The genes were identified by nucleotide BLAST search of the TSA (Transcriptome Shotgun Assembly) database of *Petunia exserta* in the NCBI with *PhSPL* genes and confirmed by alignments with AlignX program in Vector NTI Advance v11.5.2. ‘–’ indicated no transcripts was identified. Partial represent the ORF sequence was not complete [57]. (DOCX 14 kb)

Additional file 4: The analysis of nucleotide differences and the resultant amino acid variations between *PhSPL* genes and their orthologs in *P. axillaris* N, *P. inflata* S6, *P. intergifolia* and *P. exserta*. ‘/’ indicates no ortholog was identified in this species; ‘-’ indicates no protein can be translated for this gene. (DOCX 16 kb)

Additional file 5: The specific information of conserved and potential motifs in PhSPL protein sequences predicted by MEME. (DOCX 1340 kb)

Additional file 6: Primers used in this study. **a** Primer pairs used for the cloning of complete CDS. **b** Primer pairs used for qRT-PCR and transgene RT-PCR. (DOCX 18 kb)

Additional file 7: Heat map of *PhSPL* genes expression in various tissues. Differences in gene expression are shown in color according to the scale. R, roots; S, stems; L, leaves; Ab, axillary buds; If, inflorescencess; Fb, flower buds; Fr, young fruits; Sl, young seedlings; Gs, germinating seeds; Br, bracts; Co, cotyledons. (DOCX 89 kb)

Additional file 8: Phenotypes of the transgenic Arabidopsis plants overexpressing *PhSPL9a* and *PhSPL9b* genes. 32 T_2_ transgenic plants of putative single-copy transgenic lines were chosen to record flowering time and floral phenotype. Values are mean ± SD (*n* = 32). Asterisk indicated significant difference in comparison with the empty plasmid transgenic Col-0 plants (CK) (*P < 0.05 and **P < 0.001). (DOCX 12 kb)

Additional file 9: *SPL* genes of *Nicotiana tomentosiformis* annotated in NCBI [19]. (DOCX 15 kb)

## Abbreviations

ML: maximum likelihood
NJ: Neighbor-Joining
NLS: nuclear localization signal
qPCR: real-time quantitative RT-PCR
RT-PCR: reverse transcription PCR
SBP: SQUAMOSA PROMOTER BINDING PROTEIN
*SPL*: *SQUAMOSA PROMOTER BINDING PROTEIN-LIKE*
TFs: transcription factors
UTR: untranslated region
