# Supplementary material for: Genome-wide identification and characterization of the SBP-box gene family in Petunia
Source: BMC Genomics. 2018 Mar 12;19:193. doi: 10.1186/s12864-018-4537-9 (PMC6389188; doi:10.1186/s12864-018-4537-9)
Supplement: Supplementary file 1 — Orthologous SPL genes in P. axillaris N genome. aSequence ID corresponds to annotations provided by https://solgenomics.net/organism/Petunia_axillaris/genome (v1.6.2) [50]. b The transcripts were identified by nucleotide BLAST search of the TSA (Transcriptome Shotgun Assembly) database of Petunia axillaris in the NCBI and confirmed by alignment with AlignX program in Vector NTI Advance v11.5.2 [57]. (DOCX 18 kb) [file 12864_2018_4537_MOESM1_ESM.docx]

| **Gene name** | **Sequence ID of genomic DNA^a^** | **Regions of exons (bp)** | **Gene length (bp)** | **ORF length (bp)** | **Protein length (aa)** | **Transcripts in TSA database^b^** |
| --- | --- | --- | --- | --- | --- | --- |
| *PaSPL2* | Peaxi162Scf00128 | 1317750-1317086 (665), 1316942-1316806 (137),  1316380-1316114 (267), 1314940-1314606 (335) | 3145 | 1404 | 467 | GBRU01036621.1 |
| *PaCNR* | Peaxi162Scf01100 | 206985-206696 (290), 205642-205525 (118) | 1461 | 285 | 94 | GBRU01015441.1 |
| *PaSPL3* | Peaxi162Scf00031 | 1502689-1502385 (305), 1500746-1500629 (118) | 2061 | 423 | 140 | GBRU01066869.1 |
| *PaSPL4a* | Peaxi162Scf00059 | 1120398-1120106 (293), 1117244-1117118 (127) | 3281 | 420 | 139 | GBRU01069673.1 |
| *PaSPL4b* | Peaxi162Scf00981 | 452877-453235 (359), 455178-455454 (277) | 2578 | 636 | 211 | GBRU01062522.1 |
| *PaSPL4c* | Peaxi162Scf00069 | 1677950-1678287 (338), 1678775-1678973 (199) | 1024 | 537 | 178 | GBRU01065482.1 |
| *PaSPL6a* | Peaxi162Scf00175 | 585809-585034 (776), 584387-584284 (104),  583839-583145 (695) | 2665 | 1575 | 494 | GBRU01050136.1+  GBRU01062177.1 |
| *PaSPL6b* | Peaxi162Scf00026 | 1354487-1355187 (701), 1355503-1355606 (104),  1355869-1356554 (686) | 2068 | 1491 | 496 | GBRU01030464.1 |
| *PaSPL6c* | Peaxi162Scf00740 | 354941-355610 (670), 356413-357251(839) | 2311 | 1509 | 501 | GBRU01020485.1 |
| *PaSPL6d* | Peaxi162Scf00426 | 17704-18296 (593), 18670-18761 (92),  20492-21345 (854) | 3642 | 1539 | 512 | GBRU01060284.1 |
| *PaSPL6e* | Peaxi162Scf00365 | 357849-357263 (587), 356769- 356633 (137),  355991-355189 (803) | 2661 | 1527 | 508 | GBRU01067342.1 |
| *PaSPL7* | Peaxi162Scf00095 | 68208-68776 (569), 70076-70251 (176),  70765-70973 (209), 71064-71138 (75),  71236-71343 (108), 71703-71820 (118),  73286-73421 (136), 75230-75389 (160),  76323-76892 (570), 77410-77694 (285) | 9487 | 2406 | 801 | GBRU01027404.1 |
| *PaSPL8* | Peaxi162Scf00001 | 5442712-5442054 (659), 5441464-5441304 (161),  5441169-5441075 (95) | 1638 | 915 | 304 | GBRU01066837.1 |
| *PaSPL9a* | Peaxi162Scf00258 | 134805-134529 (277), 134200-134074 (127),  130430-130291 (140), 130178-129559 (620), | 5247 | 1164 | 387 | GBRU01036375.1 |
| *PaSPL9b* | Peaxi162Scf00003 | 4290358-4289970 (389), 4287727-4287588 (140),  4287458-4286863 (596) | 3496 | 1125 | 374 | GBRU01064541.1 |
| *PaSPL9c* | Peaxi162Scf00001 | 5372400-5372959 (560), 5373752-5373876 (125),  5375064-5375524 (461) | 3125 | 1146 | 382 | GBRU01041606.1 |
| *PaSPL12a* | Peaxi162Scf00919 | 338532-339127 (596), 339628-339809 (182),  339916-340490 (575), 341421-341495 (75),  341600-341707 (108), 341796-341916 (121),  342095-342236 (142), 342396-342546 (151),  343000-343632 (633), 343763-344200 (438) | 5669 | 3021 | 1006 | GBRU01006422.1 |
| *PaSPL12b* | Peaxi162Scf00164 | 507920-508485 (566), 509243-509424 (182),  510139-510299 (161), 510369-510770 (402),  511548-511622 (75), 511726-511833 (108),  512220-512337 (118), 512443-512584 (142),  512712-512862 (151), 513365-513979 (615),  514489-514926 (438) | 7007 | 2958 | 985 | GBRU01027598.1 |
| *PaSPL12c* | Peaxi162Scf00058 | 2071437-2071897 (461), 2073030-2073214 (185),  2073348-2073949 (602), 2074813-2074887 (75),  2074984-2075091 (108), 2075176-2075293 (118),  2075438-2075579 (142), 2075742-2075889 (148),  2078915-2079547 (633), 2079704-2080141 (438) | 8705 | 2910 | 969 | GBRU01031659.1 |
| *PaSPL12d* | Peaxi162Scf00126 | 1139042-1139637 (596), 1140184-1140365 (182),  1140786-1140949 (164), 1141022-1141426 (405),  1142883-1142957 (75), 1143063-1143170 (108),  1143680-1143797 (118), 1143914-1144055 (142),  1144141-1144291 (151), 1144817-1145440 (624),  1145728-1146189 (462) | 7148 | 3027 | 1008 | GBRU01021711.1 |
| *PaSPL13* | Peaxi162Scf00128 | 1476180-1475978 (203), 1474928-1474792 (137),  1474652-1474003 (650) | 2178 | 990 | 329 | GBRU01049504.1 |
