# Supplementary material for: Genome-wide identification and characterization of the SBP-box gene family in Petunia
Source: BMC Genomics. 2018 Mar 12;19:193. doi: 10.1186/s12864-018-4537-9 (PMC6389188; doi:10.1186/s12864-018-4537-9)
Supplement: Supplementary file 2 — Orthologous SPL genes in P. inflata S6 genome and P. intergifolia transcriptome. a Sequence ID corresponds to annotations provided by https://solgenomics.net/organism/Petunia_axillaris/genome (v1.0.1) [50] b The transcripts were identified by nucleotide BLAST search of the TSA (Transcriptome Shotgun Assembly) database of Petunia integrifolia (GBRV) and P. integrifolia Subsp. inflata (GBDS) in the NCBI and confirmed by alignment with AlignX program in Vector NTI Advance v11.5.2 [57]. ‘/’ indicates no orthologous transcript was found. (DOCX 22 kb) [file 12864_2018_4537_MOESM2_ESM.docx]

| **Gene name** | **Sequence ID of genomic DNA^a^** | **Regions of exons (bp)** | **Gene length (bp)** | **ORF length (bp)** | **Protein length (aa)** | **Transcripts in**  **TSA database^b^** |
| --- | --- | --- | --- | --- | --- | --- |
| *PiSPL2* | [Peinf101Scf00889](https://solgenomics.net/tools/blast/show_match_seq.pl?blast_db_id=276;id=Peinf101Scf00889;hilite_coords=580801-581139) | 580276-580940 (665), 581058-581194 (137), 581589-581852 (264), 582937-583271 (335) | 2996 | 1401 | 466 | GBRV01014410.1;  [GBDS01015263.1](https://www.ncbi.nlm.nih.gov/nuccore/GBDS01028970)+  [GBDS01006993.1](https://www.ncbi.nlm.nih.gov/nuccore/GBDS01006993) |
| *PiCNR* | [Peinf101Scf01732](https://solgenomics.net/tools/blast/show_match_seq.pl?blast_db_id=276;id=Peinf101Scf01732;hilite_coords=706785-706925) | 706690-706924 (235), 707979-708096 (118) | 1407 | - | - | GBRV01033146.1;  JI367699.1 |
| *PiSPL3* | [Peinf101Scf01026](https://solgenomics.net/tools/blast/show_match_seq.pl?blast_db_id=276;id=Peinf101Scf01026;hilite_coords=770221-770364) | 770529-770225 (305), 769027-768910 (118) | 1620 | 423 | 140 | GBRV01091560.1;  [GBDS01019446.1](https://www.ncbi.nlm.nih.gov/nuccore/GBDS01019446) |
| *PiSPL4a* | [Peinf101Scf00168](https://solgenomics.net/tools/blast/show_match_seq.pl?blast_db_id=276;id=Peinf101Scf00168;hilite_coords=637328-637480) | 637633-637341 (293), 635423-635297 (127) | 2337 | 420 | 139 | [/](https://www.ncbi.nlm.nih.gov/nuccore/GBRV01101275);  GBDS01016554.1 |
| *PiSPL4b* | [Peinf101Scf01077](https://solgenomics.net/tools/blast/show_match_seq.pl?blast_db_id=276;id=Peinf101Scf01077;hilite_coords=446509-446664) | 446883-446525 (359), 444472-444199 (274) | 2685 | 633 | 210 | GBRV01101276.1;  [GBDS01012464.1](https://www.ncbi.nlm.nih.gov/nuccore/GBDS01012464) |
| *PiSPL4c* | [Peinf101Scf02138](https://solgenomics.net/tools/blast/show_match_seq.pl?blast_db_id=276;id=Peinf101Scf02138;hilite_coords=201584-201727) | 201386-201723 (338), 202320-202518 (199) | 1133 | 537 | 178 | GBRV01093002.1;  [GBDS01015888.1](https://www.ncbi.nlm.nih.gov/nuccore/GBDS01015888) |
| *PiSPL6a* | [Peinf101Scf01278](https://solgenomics.net/tools/blast/show_match_seq.pl?blast_db_id=276;id=Peinf101Scf01278;hilite_coords=708213-708356) | 707577-708352 (776), 709077-709180 (104), 709625-710319 (695) | 2743 | 1575 | 524 | GBRV01051822.1+  GBRV01008743.1;  [GBDS01043776.1](https://www.ncbi.nlm.nih.gov/nuccore/GBDS01043776)+  [GBDS01048781.1](https://www.ncbi.nlm.nih.gov/nuccore/GBDS01048781)+  [GBDS01032796.1](https://www.ncbi.nlm.nih.gov/nuccore/GBDS01032796) |
| *PiSPL6b* | [Peinf101Scf00488](https://solgenomics.net/tools/blast/show_match_seq.pl?blast_db_id=276;id=Peinf101Scf00488;hilite_coords=431672-431815) | 431111-431811 (701), 432128-432231 (104), 432494-433179 (686) | 2069 | 1491 | 496 | /;  / |
| *PiSPL6c* | [Peinf101Scf00500](https://solgenomics.net/tools/blast/show_match_seq.pl?blast_db_id=276;id=Peinf101Scf00500;hilite_coords=355433-355576) | 354941-355575 (635), 355840-355928 (89), 356413-357251 (839) | 2311 | 1563 | 520 | GBRV01069123.1;  / |
| *PiSPL6d* | [Peinf101Scf01556](https://solgenomics.net/tools/blast/show_match_seq.pl?blast_db_id=276;id=Peinf101Scf01556;hilite_coords=171443-171583) | 170993-171582 (590), 171902-171993 (92), 173688-174541 (854) | 3549 | 1536 | 511 | GBRV01078990.1;  GBDS01049809.1+[GBDS01024183.1](https://www.ncbi.nlm.nih.gov/nuccore/GBDS01024183) |
| *PiSPL6e* | [Peinf101Scf00239](https://solgenomics.net/tools/blast/show_match_seq.pl?blast_db_id=276;id=Peinf101Scf00239;hilite_coords=103713-103856) | 104303-103717 (587), 103243-103107 (137), 102465-101666 (800) | 2638 | 1524 | 507 | GBRV01094874.1;  JI383591.1 |
| *PiSPL7* | [Peinf101Scf00150](https://solgenomics.net/tools/blast/show_match_seq.pl?blast_db_id=276;id=Peinf101Scf00150;hilite_coords=713450-713599) | 714028-713460 (569), 712157-711982(176), 711474-711263 (212), 711167-711093(75), 710995-710888 (108), 710519-710402 (118), 709123-708988 (136), 707191-707032 (160), 706091-705492 (600), 704963-704679 (285) | 9350 | 2439 | 812 | GBRV01019558.1;  GBDQ01011414.1+  [GBDS01010744.1](https://www.ncbi.nlm.nih.gov/nuccore/GBDS01010744)+  GBDQ01009682.1 |
| *PiSPL8* | [Peinf101Scf00883](https://solgenomics.net/tools/blast/show_match_seq.pl?blast_db_id=276;id=Peinf101Scf00883;hilite_coords=581899-582042) | 582561-581903 (659), 581307-581147 (161), 581021-580927 (95) | 1635 | 915 | 304 | GBRV01099512.1;  [GBDS01037829.1](https://www.ncbi.nlm.nih.gov/nuccore/GBDS01037829) |
| *PiSPL9a* | [Peinf101Scf00835](https://solgenomics.net/tools/blast/show_match_seq.pl?blast_db_id=276;id=Peinf101Scf00835;hilite_coords=598016-598141) | 598677-598413 (265), 98143-598017 (127), 593620-593481 (140), 593366-592750 (617) | 5928 | 1149 | 382 | [GBRV01070719.1](https://www.ncbi.nlm.nih.gov/nuccore/GBRV01070719);  GBDS01017339.1+ GBDS01025219.1 |
| *PiSPL9b* | [Peinf101Scf00049](https://solgenomics.net/tools/blast/show_match_seq.pl?blast_db_id=276;id=Peinf101Scf00049;hilite_coords=136396-136536) | 136147-136535 (389), 138712-138851 (140), 138963-139558 (596) | 3412 | 1125 | 374 | GBRV01101353.1;  / |
| *PiSPL9c* | [Peinf101Scf00883](https://solgenomics.net/tools/blast/show_match_seq.pl?blast_db_id=276;id=Peinf101Scf00883;hilite_coords=531984-532127) | 531564-532123 (560), 532943-533067 (125), 534495-534955 (461) | 3392 | 1146 | 381 | GBRV01100031.1;  JI377700.1 |
| *PiSPL12a* | [Peinf101Scf00197](https://solgenomics.net/tools/blast/show_match_seq.pl?blast_db_id=276;id=Peinf101Scf00197;hilite_coords=336995-337138) | 337594-336999 (596), 336483-336302 (182), 336194-335620 (575), 334685-334611 (75),  334506-334399 (108), 334310-334190 (121), 334011-333870 (142), 333713-333563 (151), 332859-332227 (633), 332096-331659 (438) | 5936 | 3021 | 1006 | GBRV01041705.1+  GBRV01000051.1;  [GBDS01033179.1](https://www.ncbi.nlm.nih.gov/nuccore/GBDS01033179)+  [GBDS01031150.1](https://www.ncbi.nlm.nih.gov/nuccore/GBDS01031150)+  GBDS01049288.1+  GBDS01016923.1+  [GBDS01014865.1](https://www.ncbi.nlm.nih.gov/nuccore/GBDS01014865) |
| *PiSPL12b* | [Peinf101Scf00437](https://solgenomics.net/tools/blast/show_match_seq.pl?blast_db_id=276;id=Peinf101Scf00437;hilite_coords=1430309-1430452) | 1429886-1430448 (563), 1431180-1431361 (182),  1432081-1432241 (161), 1432311-1432712 (402),  1433511-1433585 (75), 1433690-1433797 (108),  1434184-1434301 (118), 1434407-1434548 (142),  1434674-1434824 (151), 1435365-1435979 (615),  1436341-1436778 (438) | 6893 | 2955 | 984 | GBRV01076694.1;  JI380985.1 |
| *PiSPL12c* | [Peinf101Scf00038](https://solgenomics.net/tools/blast/show_match_seq.pl?blast_db_id=276;id=Peinf101Scf00038;hilite_coords=178383-178523) | 178892-178384 (509), 177154-176970 (185), 176836-176235(602), 175380-175306 (75), 175209-175102 (108), 175012-174895 (118), 174743-174602 (142), 174439-174292 (148), 172551-171919 (633), 171762-171325 (438) | 7568 | 2958 | 985 | GBRV01078363.1+  [GBRV01036828.1](https://www.ncbi.nlm.nih.gov/nuccore/GBRV01000048);  [GBDQ01019097.1](https://www.ncbi.nlm.nih.gov/nuccore/GBDQ01019097)+  [GBDS01015274.1](https://www.ncbi.nlm.nih.gov/nuccore/GBDS01015274)+  GBDS01044948.1+  GBDS01020650.1 |
| *PiSPL12d* | [Peinf101Scf02665](https://solgenomics.net/tools/blast/show_match_seq.pl?blast_db_id=276;id=Peinf101Scf02665;hilite_coords=151937-152080) | 151481-152076 (596), 152335-152516 (182),  154137-154300 (164), 154373-154777 (405), 156031-156105 (75), 156211-156318 (108), 156835-156952 (118), 157069-157210 (142), 157296-157446 (151), 157902-158525 (624), 158828-159289 (462) | 7809 | 3027 | 1008 | GBRV01006910.1;  [GBDS01011416.1](https://www.ncbi.nlm.nih.gov/nuccore/GBDS01011416)+  [GBDS01030685.1](https://www.ncbi.nlm.nih.gov/nuccore/GBDS01030685)+  [GBDS01037458.1](https://www.ncbi.nlm.nih.gov/nuccore/GBDS01037458)+  [GBDS01002624.1](https://www.ncbi.nlm.nih.gov/nuccore/GBDS01002624) |
| *PiSPL13* | [Peinf101Scf00889](https://solgenomics.net/tools/blast/show_match_seq.pl?blast_db_id=276;id=Peinf101Scf00889;hilite_coords=412704-412865) | 412641-412843 (203), 413842-413978 (137), 414126-414775 (650) | 2135 | 990 | 329 | GBRV01047246.1;  JI370130.1 |
