# Supplementary material for: Genome-wide identification and characterization of the SBP-box gene family in Petunia
Source: BMC Genomics. 2018 Mar 12;19:193. doi: 10.1186/s12864-018-4537-9 (PMC6389188; doi:10.1186/s12864-018-4537-9)
Supplement: Supplementary file 3 — Orthologous SPL genes in the transcriptome database of P. exserta [51]. The genes were identified by nucleotide BLAST search of the TSA (Transcriptome Shotgun Assembly) database of Petunia exserta in the NCBI with PhSPL genes and confirmed by alignments with AlignX program in Vector NTI Advance v11.5.2. ‘–’ indicated no transcripts was identified. Partial represent the ORF sequence was not complete [57]. (DOCX 14 kb) [file 12864_2018_4537_MOESM3_ESM.docx]

| **Gene name** | **Sequence ID** | **The length of ORFs (bp)** | **The length of putative proteins (amino acid)** |
| --- | --- | --- | --- |
| *PeCNR* | [-](https://www.ncbi.nlm.nih.gov/nuccore/GBRT01046073) | - | - |
| *PeSPL2* | [GBRT01041627.1](https://www.ncbi.nlm.nih.gov/nuccore/GBRT01041627) | 1401 | 466 |
| *PeSPL3* | [GBRT01046073.1](https://www.ncbi.nlm.nih.gov/nuccore/GBRT01046073) | 423 | 140 |
| *PeSPL4a* | - | - | - |
| *PeSPL4b* | [GBRT01047442.1](https://www.ncbi.nlm.nih.gov/nuccore/GBRT01047442) | 633 | 210 |
| *PeSPL4c* | [GBRT01047133.1](https://www.ncbi.nlm.nih.gov/nuccore/GBRT01047133) | 537 | 178 |
| *PeSPL6a* | [GBRT01021238.1](https://www.ncbi.nlm.nih.gov/nuccore/GBRT01021238) | 1575 | 524 |
| *PeSPL6b* | [GBRT01034463.1](https://www.ncbi.nlm.nih.gov/nuccore/GBRT01034463) | 1491 | 496 |
| *PeSPL6c* | [GBRT01031045.1](https://www.ncbi.nlm.nih.gov/nuccore/GBRT01031045) | 1563 | 520 |
| *PeSPL6d* | [GBRT01040436.1](https://www.ncbi.nlm.nih.gov/nuccore/GBRT01040436) | 1539 | 512 |
| *PeSPL6e* | [GBRT01046663.1](https://www.ncbi.nlm.nih.gov/nuccore/GBRT01048102)+  GBRT01048102.1 | 1527 | 508 |
| *PeSPL7* | [GBRT01007936.1](https://www.ncbi.nlm.nih.gov/nuccore/GBRT01007936) | 2406 | 801 |
| *PeSPL8* | [GBRT01048978.1](https://www.ncbi.nlm.nih.gov/nuccore/GBRT01048978) | 915 | 304 |
| *PeSPL9a* | [GBRT01040091.1](https://www.ncbi.nlm.nih.gov/nuccore/GBRT01040091) | 1167 | 388 |
| *PeSPL9b* | [GBRT01046991.1](https://www.ncbi.nlm.nih.gov/nuccore/GBRT01046991) | 1125 | 374 |
| *PeSPL9c* | [GBRT01047469.1](https://www.ncbi.nlm.nih.gov/nuccore/GBRT01047469) | 1096 (partial) | - |
| *PeSPL12a* | [GBRT01019541.1](https://www.ncbi.nlm.nih.gov/nuccore/GBRT01019541) | 3021 | 1006 |
| *PeSPL12b* | [GBRT01035723.1](https://www.ncbi.nlm.nih.gov/nuccore/GBRT01035723) | 2958 | 985 |
| *PeSPL12c* | [GBRT01003198.1](https://www.ncbi.nlm.nih.gov/nuccore/GBRT01003198) | 2910 | 969 |
| *PeSPL12d* | [GBRT01010547.1](https://www.ncbi.nlm.nih.gov/nuccore/GBRT01010547) | 3027 | 1008 |
| *PeSPL13* | [GBRT01047106.1](https://www.ncbi.nlm.nih.gov/nuccore/GBRT01047108) | 990 | 329 |
