# Supplementary material for: Genome-wide identification and characterization of the SBP-box gene family in Petunia
Source: BMC Genomics. 2018 Mar 12;19:193. doi: 10.1186/s12864-018-4537-9 (PMC6389188; doi:10.1186/s12864-018-4537-9)
Supplement: Supplementary file 4 — The analysis of nucleotide differences and the resultant amino acid variations between PhSPL genes and their orthologs in P. axillaris N, P. inflata S6, P. intergifolia and P. exserta. ‘/’ indicates no ortholog was identified in this species; ‘-’ indicates no protein can be translated for this gene. (DOCX 16 kb) [file 12864_2018_4537_MOESM4_ESM.docx]

| **Genes of *P. hybrida* line W115** | **Orthologs in *P. axillaris*** | **Nucleotide differences** | **Amino acid variations** | **Orthologs in *P. inflata*** | **Nucleotide differences** | **Amino acid variations** | **Orthologs in *P. integrifolia*** | **Nucleotide differences** | **Amino acid variations** | **Orthologs in *P. exserta*** | **Nucleotide differences** | **Amino acid variations** |
| --- | --- | --- | --- | --- | --- | --- | --- | --- | --- | --- | --- | --- |
| *PhCNR* | *PaCNR* | 0 | 0 | *PiCNR* | 66 | - | *PintCNR* | 66 | - | */* | / | / |
| *PhSPL2* | *PaSPL2* | 6 | 3 | *PiSPL2* | 32 | 14 | *PintSPL2* | 17 | 9 | *PeSPL2* | 10 | 3 |
| *PhSPL3* | *PaSPL3* | 0 | 0 | *PiSPL3* | 2 | 0 | *PintSPL3* | 2 | 0 | *PeSPL3* | 2 | 2 |
| *PhSPL4a* | *PaSPL4a* | 2 | 0 | *PiSPL4a* | 8 | 3 | */* | / | / | */* | / | / |
| *PhSPL4b* | *PaSPL4b* | 3 | 1 | *PiSPL4b* | 13 | 7 | *PintSPL4b* | 13 | 7 | *PeSPL4b* | 4 | 2 |
| *PhSPL4c* | *PaSPL4c* | 1 | 0 | *PiSPL4c* | 19 | 10 | *PintSPL4c* | 15 | 6 | *PeSPL4c* | 0 | 0 |
| *PhSPL6a* | *PaSPL6a* | 0 | 0 | *PiSPL6a* | 57 | 30 | *PintSPL6a* | 58 | 31 | *PeSPL6a* | 3 | 2 |
| *PhSPL6b* | *PaSPL6b* | 1 | 0 | *PiSPL6b* | 23 | 16 | */* | / | / | *PeSPL6b* | 1 | 0 |
| *PhSPL6c* | *PaSPL6c* | 66 | 30 | *PiSPL6c* | 97 | 55 | *PintSPL6c* | 101 | 60 | *PeSPL6c* | 28 | 21 |
| *PhSPL6d* | *PaSPL6d* | 0 | 0 | *PiSPL6d* | 55 | 33 | *PintSPL6d* | 54 | 36 | *PeSPL6d* | 3 | 2 |
| *PhSPL6e* | *PaSPL6e* | 1 | 0 | *PiSPL6e* | 52 | 28 | *PintSPL6e* | 48 | 25 | *PeSPL6e* | 2 | 1 |
| *PhSPL7* | *PaSPL7* | 0 | 0 | *PiSPL7* | 87 | 33 | *PintSPL7* | 74 | 30 | *PeSPL7* | 3 | 0 |
| *PhSPL8* | *PaSPL8* | 2 | 1 | *PiSPL8* | 13 | 2 | *PintSPL8* | 11 | 2 | *PeSPL8* | 2 | 1 |
| *PhSPL9a* | *PaSPL9a* | 2 | 2 | *PiSPL9a* | 42 | 18 | *PintSPL9a* | 34 | 14 | *PeSPL9a* | 10 | 5 |
| *PhSPL9b* | *PaSPL9b* | 8 | 5 | *PiSPL9b* | 26 | 11 | *PintSPL9b* | 29 | 13 | *PeSPL9b* | 35 | 6 |
| *PhSPL9c* | *PaSPL9c* | 4 | 3 | *PiSPL9c* | 26 | 15 | *PintSPL9c* | 26 | 16 | *PeSPL9c* | >4 | >4 |
| *PhSPL12a* | *PaSPL12a* | 0 | 0 | *PiSPL12a* | 27 | 8 | *PintSPL12a* | 26 | 9 | *PeSPL12a* | 5 | 2 |
| *PhSPL12b* | *PaSPL12b* | 0 | 0 | *PiSPL12b* | 84 | 40 | *PintSPL12b* | >81 | >39 | *PeSPL12b* | 3 | 1 |
| *PhSPL12c* | *PaSPL12c* | 0 | 0 | *PiSPL12c* | 110 | 45 | *PintSPL12c* | 109 | 45 | *PeSPL12c* | 4 | 3 |
| *PhSPL12d* | *PaSPL12d* | 1 | 1 | *PiSPL12d* | 88 | 33 | *PintSPL12d* | 84 | 32 | *PeSPL12d* | 7 | 5 |
| *PhSPL13* | *PaSPL13* | 6 | 2 | *PiSPL13* | 9 | 2 | *PintSPL13* | 4 | 1 | *PeSPL13* | 4 | 3 |
