# Supplementary material for: Genome-wide identification and characterization of the SBP-box gene family in Petunia
Source: BMC Genomics. 2018 Mar 12;19:193. doi: 10.1186/s12864-018-4537-9 (PMC6389188; doi:10.1186/s12864-018-4537-9)
Supplement: Supplementary file 5 — The specific information of conserved and potential motifs in PhSPL protein sequences predicted by MEME. (DOCX 1340 kb) [file 12864_2018_4537_MOESM5_ESM.docx]

motif1


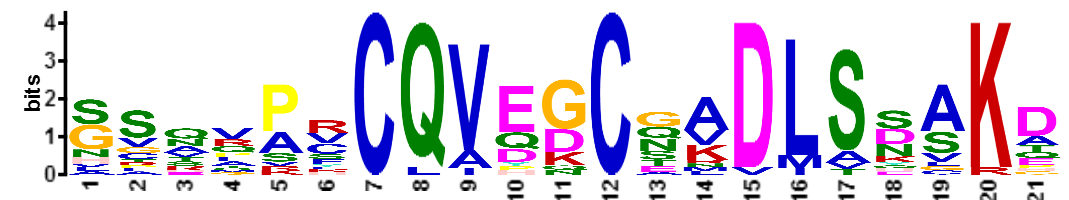


motif2


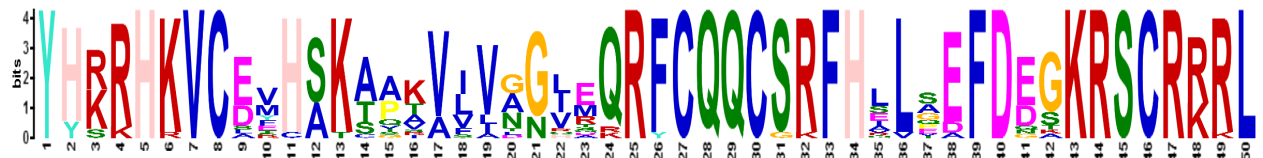


motif3


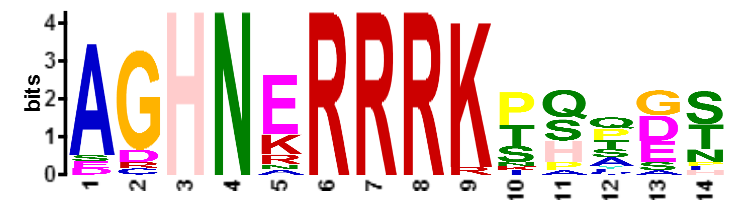


motif4


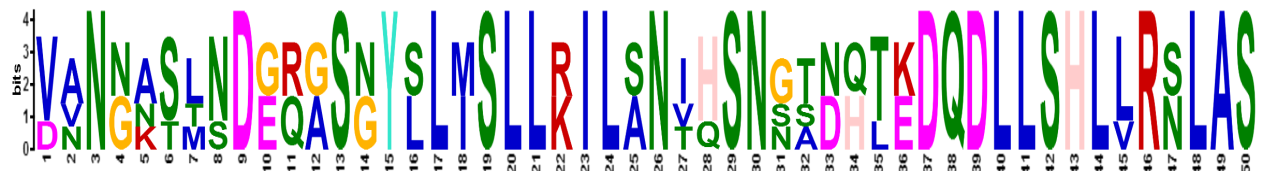


motif5


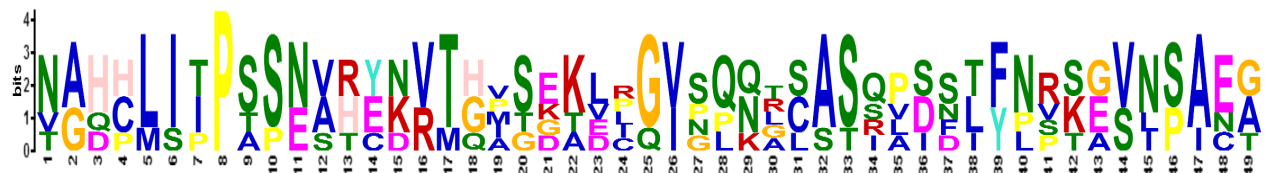


motif6


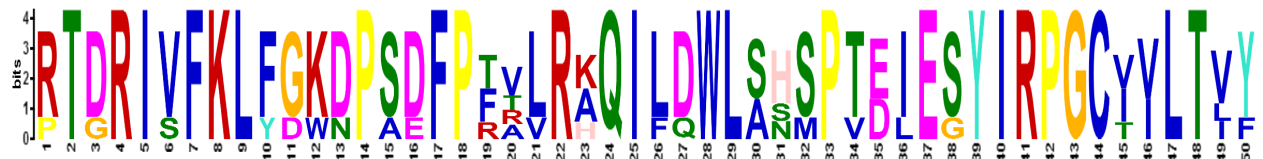


motif7


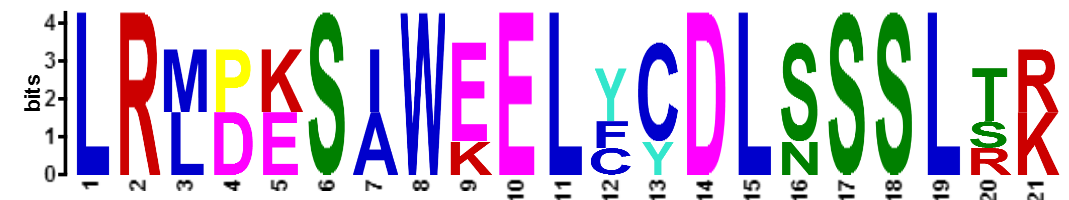


motif8


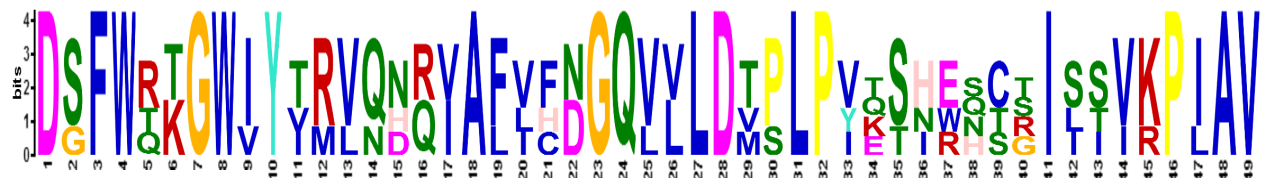


motif9


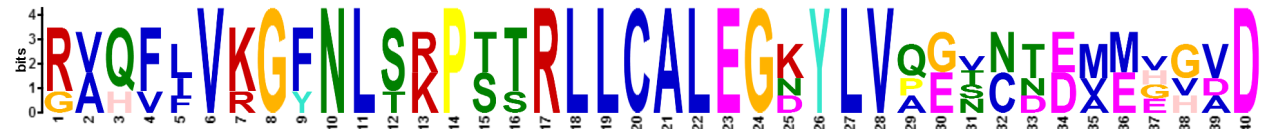


motif10


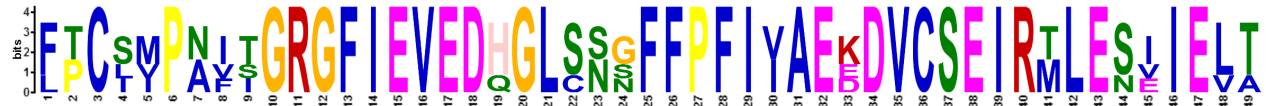


motif11


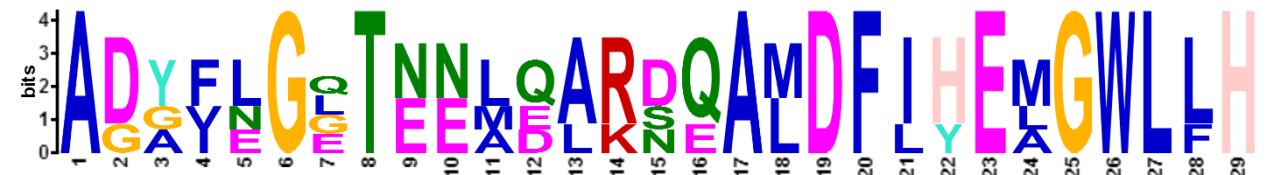


motif12


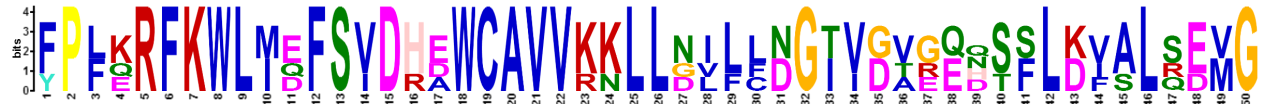


motif13


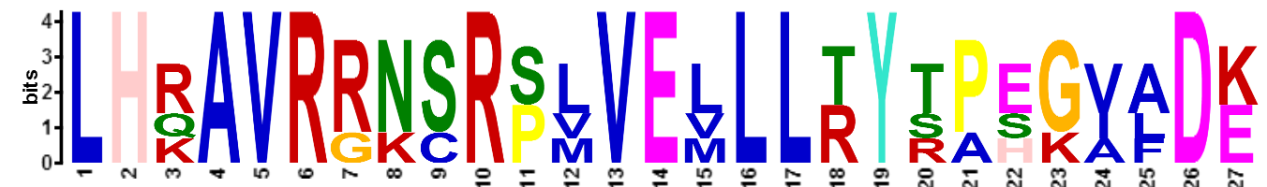


motif14


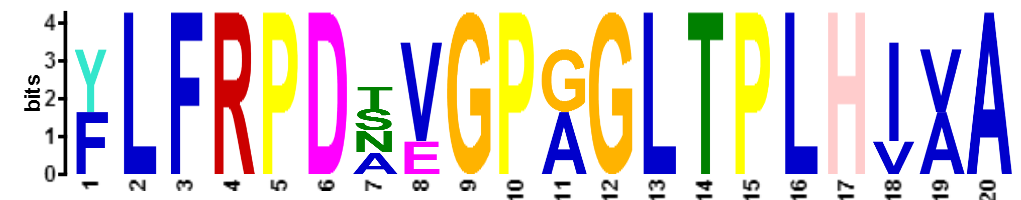


motif15


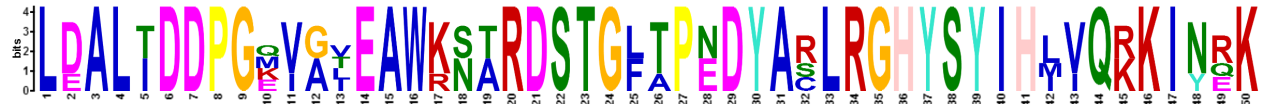


motif16


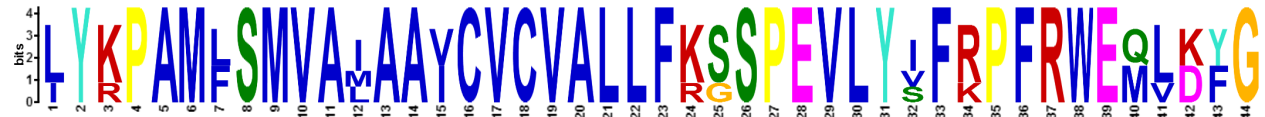


motif17


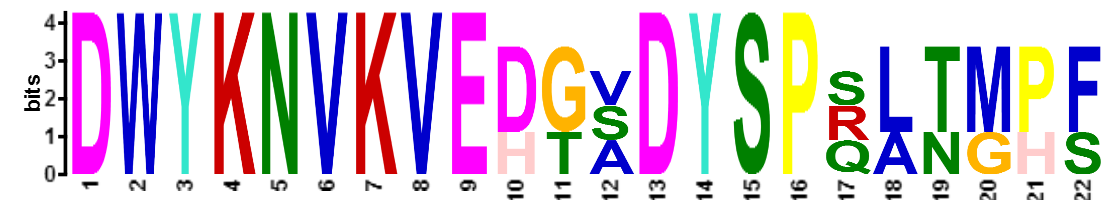


motif18


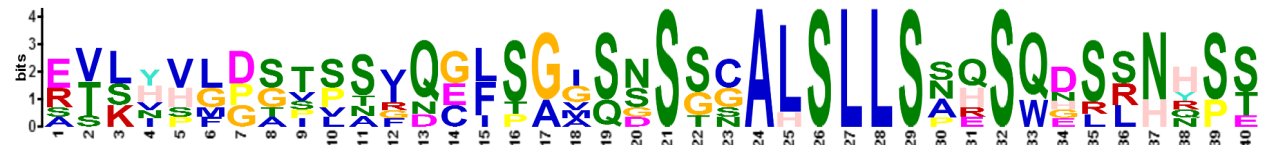


motif19


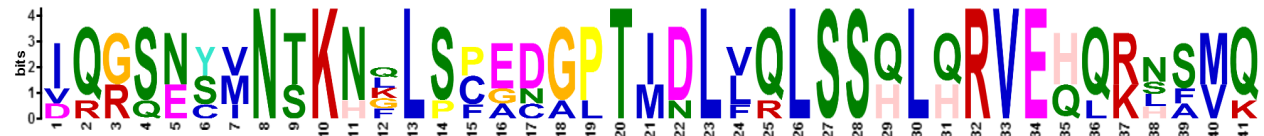


Motif20

**
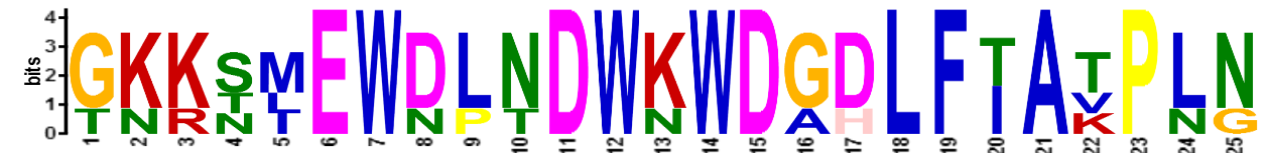
**
