# Supplementary material for: Genome-wide identification and characterization of the SBP-box gene family in Petunia
Source: BMC Genomics. 2018 Mar 12;19:193. doi: 10.1186/s12864-018-4537-9 (PMC6389188; doi:10.1186/s12864-018-4537-9)
Supplement: Supplementary file 6 — Primers used in this study. a Primer pairs used for the cloning of complete CDS. b Primer pairs used for qRT-PCR and transgene RT-PCR. (DOCX 18 kb) [file 12864_2018_4537_MOESM6_ESM.docx]

**a**

| **Primers** | **Sequence (5’ to 3’)** |
| --- | --- |
| *PhCNR(fw)*  *PhCNR(rev)* | TTTCAAGTTTCACACTCTTTCATCC  CGTTCTCCCTTATTCATAAGGAAATG |
| *PhSPL2(fw)*  *PhSPL2(rev)* | AGATGGAGTGGAATGTGAAGTG  AGCTCAACAGGTCTTGAAATCC |
| *PhSPL3(fw)*  *PhSPL3(rev)* | CATCATTCTCATCTTCTTTGTACTCC  TTGGTTAAAGGGAAATTTGGCAATG |
| *PhSPL4a(fw)*  *PhSPL4a(rev)* | CACTTCCTCGCAAGCCTATA  GGAACAGTGGATTGGTGATTAC |
| *PhSPL4b(fw)*  *PhSPL4b(rev)* | CCCACTTCATCATCACATAACCTA  TAGAACATAGGAGAATGACAGAAGAG |
| *PhSPL4c(fw)*  *PhSPL4c(rev)* | AAGCATGGCAACCTACAACC  TCACTATTAACTAGAACCACACGTA |
| *PhSPL6a(fw)*  *PhSPL6a(rev)* | GCACTTGTTATTGGATGGAACCT  AAGAACCTTGTAGCTCTTCATAACC |
| *PhSPL6b(fw)*  *PhSPL6b(rev)* | CTAGCAATGGAACCTATGAACTATG  TCGTTGCCTCTTGATTGTTGG |
| *PhSPL6c(fw)*  *PhSPL6c(rev)* | AATGGAATCTTGGAGCTTTGTCT  TAGAAACTTTCATGCTGGTAATGC |
| *PhSPL6d(fw)*  *PhSPL6d(rev)* | GGGTTTGTATGAAATTGGTGAAAGA  TTTGATGTCAGTCCAGATATTTGC |
| *PhSPL6e(fw)*  *PhSPL6e(rev)* | AATGGAGTCTTGGAGCTATGTGT  TGCTCAAGTGATCCAGAATTGC |
| *PhSPL7(fw1)*  *PhSPL7(rev1)*  *PhSPL7(fw2)*  *PhSPL7(rev2)* | CAACCCTCCCAATTTCTAGTAG  GCGAAATACCATGTTGTTGAG  AAGACCCAGCTGTACACCTATATG  GAAAGTTCTTCTGGTTAAGTGTTGC |
| *PhSPL8(fw)*  *PhSPL8(rev)* | GTGGAGCAGATCAAAGTGAAAGA  GTAGAGCAACAGCAGCATACAA |
| *PhSPL9a(fw)*  *PhSPL9a(rev)* | GTGATGAATGGGCTCAGCAACTCA  AGTCTTCCTACGACGATGATATAGT |
| *PhSPL9b(fw)*  *PhSPL9b(rev)* | GTAATGAATAGGCACTTGACGTCAG  GGCCATTCGTCGATAGAGCC |
| *PhSPL9c(fw)*  *PhSPL9c(rev)* | CTACACATAAACAGGCAAATGG  GGAAGCAAATTATCGACAAGAG |
| *PhSPL12a(fw1)*  *PhSPL12a(rev1)*  *PhSPL12a(fw2)*  *PhSPL12a(rev2)* | TGAGCTACACGCGAGGAGAC  GGTTCTGCACCCTAATGTAAATC  GTTATGACCTGAGCTCCAGTTTG  AATCAGCTTGTTCCGAAGTCCA |
| *PhSPL12b(fw1)*  *PhSPL12b(rev1)*  *PhSPL12b(fw2)*  *PhSPL12b(rev2)* | GATGGAGGCACATAATTGTAATGG  CCACTCGATCATTTACCCTAG  AGGAAGCTTCTTAATGCATC  TTCAGAAATGGTCTCCCTAAGAATC |
| *PhSPL12c(fw1)*  *PhSPL12c(rev1)*  *PhSPL12c(fw2)*  *PhSPL12c(rev2)* | CTAACTTTATCGGAAACACCACTT  AGCATAACGTAAATCCATCC  GATCTGAGCTCCAGCTTGAC  TGTACTATCAGATTTATTGGGAGGT |
| *PhSPL12d(fw1)*  *PhSPL12d(rev1)*  *PhSPL12d(fw2)*  *PhSPL12d(rev2)* | CCCACCTTAATTTATTGGAAGTCTT  CCATCCGAAGATATATCGTC  TCTGGATTGGTTAGCACACA  GGCATTTCTACACCACTCTTCTA |
| *PhSPL13(fw)*  *PhSPL13(rev)* | GCAATTGGAAGGTTCTTATTGTATG  AGTTGGATAAGCTGAAAGTTTAGTG |

**b**

| **Primers** | **Sequence (5’ to 3’)** | **Description** |
| --- | --- | --- |
| *PhCNR(fw)*  *PhCNR(rev)* | TGGAAAAAGTTCAAGTTGAAGCAGC  CGTTCTCCCTTATTCATAAGGAAATG | qRT-PCR |
| *PhSPL2(fw)*  *PhSPL2(rev)* | CTGCAGCCGAGGTTTTCAATC  TATTAGCAGCCAATGTATGGTTTCG | qRT-PCR |
| *PhSPL3(fw)*  *PhSPL3(rev)* | GTTCATGTGATTCTCCTGGAGAAGG  TGGTTAAAGGGAAATTTGGCAA | qRT-PCR |
| *PhSPL4a(fw)*  *PhSPL4a(rev)* | GTTTGATGAATCCAAAAGGAGTTGC  GGAACAGTGGATTGGTGATTAC | qRT-PCR |
| *PhSPL4b(fw)*  *PhSPL4b(rev)* | GTCTAATGGTACTATTGCAGAAGGG  GAAATAGAACATAGGAGAATGACAG | qRT-PCR |
| *PhSPL4c(fw)*  *PhSPL4c(rev)* | TTGAAGATAACCAATGCAGACAGAT  CTAGAACCACACGTAATAACGAAGG | qRT-PCR |
| *PhSPL5(fw)*  *PhSPL5(rev)* | TTGTCAACAGTGTAGCAGATCGTCA  AAACTCACTCACTGAATGAAACCTG | qRT-PCR |
| *PhSPL6a(fw)*  *PhSPL6a(rev)* | CCCCTGTAAAAAACATCCAGCATC  GGAGTTGGTGAAATGTCTGTTGAAT | qRT-PCR |
| *PhSPL6b(fw)*  *PhSPL6b(rev)* | AGAAGCAGTCACCAACAAAAAACC  GCGCAATGATTTTCTGAGTGGT | qRT-PCR |
| *PhSPL6c(fw)*  *PhSPL6c(rev)* | TCTTGCACGTTCTAGACTCAACTTC  CAACTCCCAGAAGCTTTTCAGATAT | qRT-PCR |
| *PhSPL6d(fw)*  *PhSPL6d(rev)* | CTATCAGGGATATCAAGCTCTAGCAA  GGAATTAGTATCTGTTCCAGTCCACC | qRT-PCR |
| *PhSPL6e(fw)*  *PhSPL6e(rev)* | GAGGTTCAGATTTTGTTTCCCGT  AGGTTTACCCCTGGTAACTTTTCAG | qRT-PCR |
| *PhSPL7(fw)*  *PhSPL7(rev)* | AAATCTTTCAGTGGTTGGCCAGTAT  TCTAGTAACTTACCCCACCTGAATG | qRT-PCR |
| *PhSPL8(fw)*  *PhSPL8(rev)* | CACTCATGATCAAAACACATCTCTAAT  TTTGGCACAACCATGAACCC | qRT-PCR |
| *PhSPL9a(fw)*  *PhSPL9a(rev)* | AAACTGCTTCACTGGAGTCTCAGAT  TTACGAGGCATGTCATGTGAAGTG | qRT-PCR and transgene RT-PCR |
| *PhSPL9b(fw)*  *PhSPL9b(rev)* | TTCCTTCAGGAGACTGTTTCCCT  CTGCTGCTAGCTTCATTTCCTTTAA | qRT-PCR and transgene RT-PCR |
| *PhSPL12a(fw)*  *PhSPL12a(rev)* | CTGTACAAGCCTGCAATGTTTTCG  AATCAGCTTGTTCCGAAGTCCA | qRT-PCR |
| *PhSPL12b(fw)*  *PhSPL12b(fw)* | TCCTCACTTGCATTATACAAACCTG  TTCAGAAATGGTCTCCCTAAGAATC | qRT-PCR |
| *PhSPL12c(fw)*  *PhSPL12c(rev)* | AAGCTGGCTTATGGAAGTCGG  AGATTTATTGGGAGGTAAAATCAGC | qRT-PCR |
| *PhSPL12d(fw)*  *PhSPL12d(fw)* | AACATCAGTTGCAATATACAAGCCG  GGCATTTCTACACCACTCTTCTA | qRT-PCR |
| *PhSPL13(fw)*  *PhSPL13(rev)* | CAGTCAAAACTCGTTTCCTGATTCT  CTGCACCATGTGACTAAAACCAA | qRT-PCR |
| *PhEF1a(fw)*  *PhEF1a(rev)* | CCTGGTCAAATTGGAAACGG  CAGATCGCCTGTCAATCTTGG | qRT-PCR and RT-PCR control |
| *AtEF1α(fw)*  *AtEF1α(rev)* | GCAAGATGGATGCCACTACCC  AGTGGGAGACGAAGGGGCT | transgene RT-PCR control |
