# Supplementary material for: Genome-wide identification and characterization of the SBP-box gene family in Petunia
Source: BMC Genomics. 2018 Mar 12;19:193. doi: 10.1186/s12864-018-4537-9 (PMC6389188; doi:10.1186/s12864-018-4537-9)
Supplement: Supplementary file 7 — Heat map of PhSPL genes expression in various tissues. Differences in gene expression are shown in color according to the scale. R, roots; S, stems; L, leaves; Ab, axillary buds; If, inflorescencess; Fb, flower buds; Fr, young fruits; Sl, young seedlings; Gs, germinating seeds; Br, bracts; Co, cotyledons. (DOCX 89 kb) [file 12864_2018_4537_MOESM7_ESM.docx]

VIII

I

II

III

IV

V

VI

VII

0.00


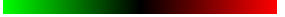


0.01

5.0E-4

R S L Ab If Fb Fr Sl Gs Br Co


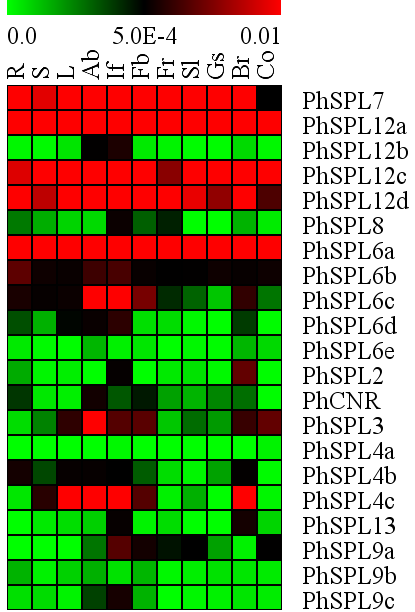


*PhSPL7*

*PhSPL12a*

*PhSPL12b*

*PhSPL12c*

*PhSPL12d*

*PhSPL8*

*PhSPL6a*

*PhSPL6b*

*PhSPL6c*

*PhSPL6d*

*PhSPL6e*

*PhSPL2*

*PhCNR*

*PhSPL3*

*PhSPL4a*

*PhSPL4b*

*PhSPL4c*

*PhSPL13*

*PhSPL9a*

*PhSPL9b*

*PhSPL9c*
