# Supplementary material for: Genome-wide identification and characterization of the SBP-box gene family in Petunia
Source: BMC Genomics. 2018 Mar 12;19:193. doi: 10.1186/s12864-018-4537-9 (PMC6389188; doi:10.1186/s12864-018-4537-9)
Supplement: Supplementary file 8 — Phenotypes of the transgenic Arabidopsis plants overexpressing PhSPL9a and PhSPL9b genes. 32 T2 transgenic plants of putative single-copy transgenic lines were chosen to record flowering time and floral phenotype. Values are mean ± SD (n = 32). Asterisk indicated significant difference in comparison with the empty plasmid transgenic Col-0 plants (CK) (*P < 0.05 and **P < 0.001). (DOCX 12 kb) [file 12864_2018_4537_MOESM8_ESM.docx]

| **Lines** | **Cauline leaves (numbers)** | **Rosette leaves (numbers)** | **Bolting time (days)** |
| --- | --- | --- | --- |
| CK | 2.3±0.5 | 12.7±0.8 | 27.7±1.5 |
| *SPL9a*-4 | 2.0±0.5 | 10.2±0.4** | 21.2±0.4** |
| *SPL9a*-6 | 2.1±0.4 | 10.2±0.6** | 24.5±0.9** |
| *SPL9a*-36 | 2.3±0.5 | 10.3±0.3** | 26.3±0.5 |
| *SPL9b*-2 | 2.2±0.3 | 10.8±0.9* | 21.6±1.8** |
| *SPL9b*-4 | 2.2±0.4 | 11.4±0.5 | 22.6±2.5** |
| *SPL9b*-27 | 2.3±0.6 | 11.5±0.5 | 25.2±1.5* |
