# Supplementary material for: Genome-wide identification and characterization of the SBP-box gene family in Petunia
Source: BMC Genomics. 2018 Mar 12;19:193. doi: 10.1186/s12864-018-4537-9 (PMC6389188; doi:10.1186/s12864-018-4537-9)
Supplement: Supplementary file 9 — SPL genes of Nicotiana tomentosiformis annotated in NCBI [19]. (DOCX 15 kb) [file 12864_2018_4537_MOESM9_ESM.docx]

| **Accession number** | **Gene ID** | **Han et al. (2016)** | **CDS length (bp)** | **Petunia orthologs** |
| --- | --- | --- | --- | --- |
| XM_009622323.2 | LOC104112407 | *NtomSPL2* | 1482 | *PhSPL2* |
| XM_009614788.2 | LOC104106284 |  | 1398 |  |
| XM_018777117.1 | LOC104114555 |  | 1152 |  |
| XM_009604018.2 | LOC104097461 | *NtomSPL3* | 417 | *PhCNR* |
| XM_009624346.2 | LOC104114007 |  | 423 | *PhSPL3* |
| XM_009622091.2 | LOC104112229 |  | 519 | *PhSPL4a* |
| XM_009627895.2 | LOC104116930 |  | 546 | *PhSPL4b* |
| XM_009597897.2 | LOC104092321 |  | 519 | *PhSPL4c* |
| XM_009617470.2 | LOC104108435 | *NtomSPL6a* | 1617 | *PhSPL6e* |
| XM_009628755.2 | LOC104117682 | *NtomSPL6c* | 1572 | *PhSPL6a* |
| XM_009602149.2 | LOC104095904 | *NtomSPL6d* | 1575 | *PhSPL6b* |
| XM_009624092.2 | LOC104113803 | *NtomSPL6e* | 1569 | *PhSPL6d* |
| XM_009611817.2 | LOC104103876 | *NtomSPL7* | 2427 | *PhSPL7* |
| XM_009595125.2 | LOC104090092 | *NtomSPL8* | 741 |  |
| XM_009609556.2 | LOC104101982 |  | 927 | *PhSPL8* |
| XM_009613814.2 | LOC104105495 | *NtomSPL9* | 1161 | *PhSPL9a* |
| XM_009588930.2 | LOC104084968 |  | 1113 | *PhSPL9b* |
| XM_009627295.2 | LOC104116444 |  | 1146 | *PhSPL9c* |
| XM_009593791.2 | LOC104088994 |  | 2997 | *PhSPL12a* |
| XM_009619233.2 | LOC104109858 |  | 2883 | *PhSPL12c* |
| XM_009619287.2 | LOC104109903 |  | 3006 | *PhSPL12b*  *PhSPL12d* |
| XM_009622742.2 | LOC104112735 | *NtomSPL13* | 981 | *PhSPL13* |
